# Dirac half-metallicity of Thin PdCl_3_ Nanosheets: Investigation of the Effects of External Fields, Surface Adsorption and Defect Engineering on the Electronic and Magnetic Properties

**DOI:** 10.1038/s41598-019-57353-3

**Published:** 2020-01-14

**Authors:** Asadollah Bafekry, Catherine Stampfl, Francois M. Peeters

**Affiliations:** 1 Department of Physics, University of Guilan, 41335-1914 Rasht, Iran; 2 Department of Physics, University of Antwerp, Groenenborgerlaan 171, B-2020 Antwerp, Belgium; 3 School of Physics, The University of Sydney, New South Wales, 2006 Australia

**Keywords:** Materials science, Materials for devices, Electronic devices

## Abstract

PdCl_3_ belongs to a novel class of Dirac materials with Dirac spin-gapless semiconducting characteristics. In this paper based, on first-principles calculations, we have systematically investigated the effect of adatom adsorption, vacancy defects, electric field, strain, edge states and layer thickness on the electronic and magnetic properties of PdCl_3_ (palladium trichloride). Our results show that when spin-orbital coupling is included, PdCl_3_ exhibits the quantum anomalous Hall effect with a non-trivial band gap of 24 meV. With increasing number of layers, from monolayer to bulk, a transition occurs from a Dirac half-metal to a ferromagnetic metal. On application of a perpendicular electrical field to bilayer PdCl_3_, we find that the energy band gap decreases with increasing field. Uniaxial and biaxial strain, significantly modifies the electronic structure depending on the strain type and magnitude. Adsorption of adatom and topological defects have a dramatic effect on the electronic and magnetic properties of PdCl_3_. In particular, the structure can become a metal (Na), half-metal (Be, Ca, Al, Ti, V, Cr, Fe and Cu with, respective, 0.72, 9.71, 7.14, 6.90, 9.71, 4.33 and 9.5 *μ*_*B*_ magnetic moments), ferromagnetic-metal (Sc, Mn and Co with 4.55, 7.93 and 2.0 *μ*_*B*_), spin-glass semiconductor (Mg, Ni with 3.30 and 8.63 *μ*_*B*_), and dilute-magnetic semiconductor (Li, K and Zn with 9.0, 9.0 and 5.80 *μ*_*B*_ magnetic moment, respectively). Single Pd and double Pd + Cl vacancies in PdCl_3_ display dilute-magnetic semiconductor characteristics, while with a single Cl vacancy, the material becomes a half-metal. The calculated optical properties of PdCl_3_ suggest it could be a good candidate for microelectronic and optoelectronics devices.

## Introduction

Over the last few years two-dimensional materials (2DM) have been attracting enormous attention because they are considered potential candidates for future applications. The prospect of the Dirac half-metal (DHM) and the alternative of the quantum anomalous Hall effect (QAHE) without external fields is a great challenge due to the structural complexities of the 2D honeycomb lattice. A significant obstacle for practical applications of the QAHE is the lack of suitable QAH materials with a large non-trivial band gap, magnetic order and high carrier mobility. Half-metallicity, together with a wide half-metallic band gap and unusual ferromagnetic character have become a key driving force to develop next-generation spintronic devices. In 2DM, the spin of electrons provide an additional degree of freedom to tune their electronic and magnetic properties^1^. Quantum anomalous Hall insulators (Chern insulators, i.e. a quantized version of QAH^2^) are a novel topological phase of matter characterized by a finite Chern number and helical edge electron states within the bulk band gap^3,4^. Without an external magnetic field, and in the presence of magnetic interactions including ferromagnetic (FM) and antiferromagnetic (AFM) order, time-reversal symmetry (TRS) is broken while opening a non-trivial spin-orbital coupling (SOC) induced gap, giving rise to a quantized anomalous Hall conductivity^5,6^. For the first time, the idea of quantized Hall conductance without Landau levels on a honeycomb lattice was introduced by Haldane^7^. Exhibiting the QAHE, in the 2D honeycomb lattice is suggested as the first ingredient^8,9^.

Recently, a variety of QAH materials based on 2D honeycomb lattices of group IV materials including silicene, germanene, and stanene, have been predicted which possess a relatively strong intrinsic SOC, by introducing an exchange interaction via magnetic adatoms and surface functionalization^10,11^. Also 2DM based on the addition of adatoms and impurities^12–15^, or chemical decorations^8,12^ of graphene-like and Bi-based materials, and also in metal organic-frameworks^16,17^, interface and heterostructure materials^18–24^, it is predicted that the QAHE could be present. Too weak electronic correlations is an important problem in these 2DM in order to drive magnetism and to break TRS. Although, 2D Kagome lattices could potentially exhibit the QAHE, the nontrivial band gaps of these materials are too small and impede the observation of QAHE^17,25,26^.

Spin-gapless semiconductors (SGS), exhibiting a band gap in one of the spin channels and a zero band gap in the other, have received considerable recent attention due to their unique electronic properties and potential applications in novel spintronic devices^27^. The DHMs are based on a combination of single-spin massless Dirac fermions and half-semimetals with broken TRS. Only a few 2DM such as YN_2_, C_7_N_6_, VCl_3_ and NiCl_3_^28–31^ show DHM characteristics.

Such materials are particularly suitable for utilization of their electronic spin degrees in nanoelectronic devices^32–35^. Accounting for SOC, the gap opening may trigger a QAH insulator transition in one spin channel only, which has been predicted for a few materials. The search for a new member of the Dirac SGS family with the QAHE is of great importance for both fundamental interest and practical applications. Also, QSHE has been reported in previously studies^25,36^. The layered crystals of transition-metal trichloride, and the family of layered materials with the general formula TMX_3_ (TM = Ti, V, Cr, Fe, Mo, Ru, Rh, Ir) and X is a halogen anion (X = Cl, Br, I) have become of interest for this purpose. In these structures, the transition-metal atoms are uniformly distributed in a honeycomb structure and the electronic and magnetic properties exhibit unusual features^30,37–39^ that have potential to be exploited in spintronic applications. These structures have been investigated for many years, before the present focus on layered materials^40^. Due to the weak interlayer van der Waals interactions, 2DM can be easily exfoliated from the corresponding 3DM by applying different technologies^41^.

Despite the fact that two-dimensional materials (2DM) hold great potential for a wide range of applications, it will be necessary to modulate their intrinsic properties. Several approaches have been developed to modify the electronic properties of 2DM. These methods involve substitutional doping, defect engineering, surface functionalization with adatoms, application of an electric field or strain, and by affecting the edge states. Many efforts have been put forth on the electronic properties and modification via these ways^42–56^. For example, with regard to achieving long-range magnetism in 2D systems, in ref. ^43^ semi (half) hydrogenated silicene was predicted to be a room-temperature ferromagnetic material, while ref. ^48^ reported a new two-dimensional honeycomb-Kagome structure which is predicted to possess the coexistence of spin-polarized multiple Dirac cones and nodal rings, with an estimated Curie temperature of 204 K. Also, Zhang *et al*.^45^ predicted from first-principles calculations that 2D SnHN and SnOH lattices possess the desirable combination of a sizeable nontrivial band-gap, high Curie temperature, and high carrier mobility. In this paper, based on first principles calculations, we investigate the electronic and magnetic properties of PdCl_3_, as an ideal candidate material for realizing both DHM and QAHE. When the SOC effect is included, a band gap opening occurs in one spin channel, which would lead towards the QAHE and PdCl_3_ becomes a Chern insulator due to TRS breaking. The physical origin of this QAH effect is due to both the intrinsic SOC and ferro-magnetism of the PdCl_3_. We comprehensively investigate the effect of layer thickness, electrical field and strain on the atomic and electronic structure of PdCl_3_. The results show that as the number of layers of PdCl_3_ is increased from monolayer to bulk, an electronic transition occurs from Dirac half-metal (monolayer) to ferromagnetic metal (quadlayer). A perpendicular electric field on the PdCl_3_ bilayer decreases the band gap. Upon uniaxial and biaxial strains, the variation in the electronic structure of PdCl_3_ can be controlled depending on the type and magnitude of the strain. The transition of electronic states in PdCl_3_ through layer thickness and electric field modulation, could shed light on the tailoring of such materials for the future development of nanoelectronic devices. Adsorption of adatoms and vacancy defects are able to further modify the electronic and magnetic properties of PdCl_3_. While pristine PdCl_3_ is a DHM, with adsorption of Al, Li, Na, K, Be, Mg, Ca, Sc, Ti, V, Cr, Mn, Fe, Co, Ni, Cu and Zn adatoms, it can be turned into a metal, half-metal, ferromagnetic-metal, spin-glass semiconductor and dilute-magnetic semiconductor.

## Method

In this work we perform total energy and electronic structure calculations using density functional theory within the generalized gradient approximation of Perdew-Burke-Ernzerhof (GGA-PBE)^57^ for the exchange-correlation functional. We use norm-conserving pseudopotentials^58^ for Pd and Cl and the other adatoms. The wave functions are expanded in a linear combination of multiple pseudoatomic orbitals (LCPAOs) generated using a confinement scheme^59,60^. The **k**-points for sampling over the Brillouin zone (BZ) integration are generated using the Monkhorst-Pack scheme^61^ where a **k**-point mesh of 17 × 17 × 1 for the primitive unit cell is used. After convergence tests in OpenMX, we choose an energy cutoff of 400 Ry, so that the total-energy converges to below 1.0 meV/atom. The geometries are fully relaxed until the force acting on each atom is less than 1 meV/Å. The PdCl_3_ structures are modeled as a periodic slab with a sufficiently large vacuum layer of 22 Å in order to avoid interaction between adjacent layers. In order to accurately describe the vdW interaction in the few-layer PdCl_3_ systems, we adopted the empirical correction method presented by Grimme (DFT-D2)^62^ which has been demonstrated as reliable for describing the long-range vdW interactions. Electron charge transfer is calculated using the Mulliken charge analysis^63^. We perform full structural optimizations, where all atoms are relaxed in all directions and calculations are carried out using 2 × 2 × 1 supercell of PdCl_3_, which contains 32 atoms (8 Pd and 24 Cl atoms). Simulated scanning tunneling microscopy (STM) images are obtained using the Tersoff-Hamann theory^64^, as implemented in the OpenMX code and are graphed using the WSxM software^65^.

Additionally, we calculate the optical properties, including dielectric function, absorption coefficient, reflectivity, refractive index and extinction coefficient of PdCl_3_ using SIESTA^66^. The exchange-correlation functional is also taken as the GGA-PBE. Core electrons are replaced by norm-conserving, nonlocal Trouiller-Martins pseudopotentials^58^. A 400 Ry mesh cut-off is chosen and self-consistent calculations are performed with a mixing parameter of 0.1. The convergence criterion for the density matrix is taken as 10^−4^ Ry.

## Pristine PdCl_3_

**Figure 1:**
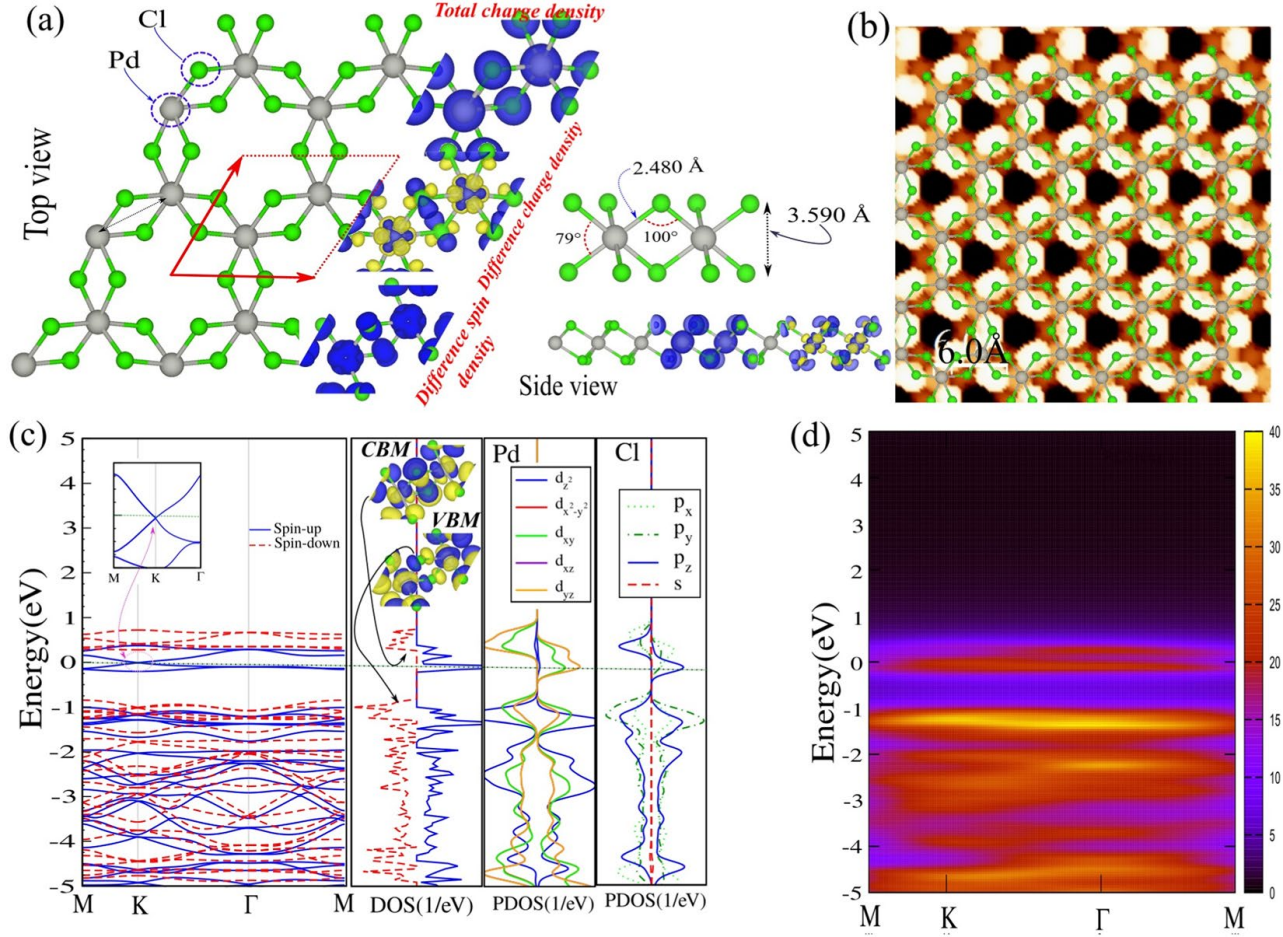
(**a**) Optimized atomic structures of the PdCl_3_ nanosheet, with its hexagonal primitive unit cell indicated by a red parallelogram. The gray (green) balls are Pd (Cl) atoms. Total, difference charge density and the difference spin density distributions are indicated in the same panel. The blue and yellow regions show, respectively, the $\uparrow$ and $\downarrow$ spin states. (**b**) Simulated STM image. The STM images is overlayed with the PdCl_3_ structure. (**c**) Band structure and corresponding DOS and PDOS. The charge densities of the VBM and CBM are indicated in the inset. (**d**) Intensity map of electronic band structure. The zero of energy is set at ${E}_{F}$.

The structure of the PdCl_3_ monolayer with the space group *P*_3*m*1_ consists of a trilayer Cl-Pd-Cl (a sheet of Pd atoms sandwiched between two sheets of Cl atoms). The Pd atoms form a 2D honeycomb lattice and each Pd atom is bonded to six Cl atoms in an octahedral environment (see Fig. 1(a)). The optimized lattice constant of PdCl_3_ is 6.50 Å. The bond lengths of Pd-Pd and Pd-Cl are, respectively, 3.81 and 2.48 Å, while the vertical distance between the two Cl atomic planes is 3.59 Å. In an earlier work^67^, DFT calculations were performed where it was reported that the ground state is a 100% spin-polarized DHM with a ferromagnetic Curie temperature of 528 K as predicted from Monte Carlo simulations. Including spinorbit coupling revealed the QAHE due to the splitting of the manifold of Pd *d*-states near the Fermi level. This study furthermore reported the dynamical stability of this structure through calculation of the phonon modes, and demonstrated the absence of imaginary modes. Thermal stability was also checked by using molecular dynamics simulations^67^.

In Fig. 1(a) we show the total and difference electron charge densities. A high charge density can be seen around the Cl atoms, reflecting the electronegative character. The Pauling electro-negativity values for Pd and Cl atoms are 2.2 and 3.16, respectively. This electro-negativity difference explains the electron transfer from Pd atoms to Cl atoms. According to the Mulliken charge analysis, each Pd atom in PdCl_3_ loses 0.53 electrons. The calculated STM image of PdCl_3_ is shown in Fig. 1(b). To produce the calculated image, the Kohn-Sham charge density is integrated to 2 eV below *E*_*F*_. In Fig. 1(b) the inset structure represents the PdCl_3_ repeating unit cell. We see that the Cl atom in one sublayer exhibits a brighter spot than another sublayer.

In order to explain the electronic states of PdCl_3_ and clarify contributions from different orbitals, we have calculated the electronic band structure and corresponding density of states (DOS) and projected PDOS, as shown in Fig. 1(c,d). The $\downarrow$ spin channel of PdCl_3_ is an indirect semiconductor and possess a 1.12 eV band gap, where the valence band maximum (VBM) and the conduction band minimum (CBM) are located at M and $\Gamma$ points, respectively. The VBM of the $\uparrow$ spin channel shifts down and crosses the *E*_*F*_ and exhibits a gapless semiconductor character with a linear band dispersion relation near *E*_*F*_. The massless Dirac fermions are found in the $\uparrow$ spin channel of PdCl_3_ at the high-symmetry K point of the Brillouin zone, where they are DHM with 100% spin polarization. Our result is in good agreement with previous calculations^67^. The electronic structure of PdCl_3_, exhibits a rather rare Dirac spin-gapless semiconductor character and unlike conventional Dirac-cone structures, here an excited Dirac fermion can be fully spin polarized (see Fig. 1(c)). From the PDOS of PdCl_3_, we see that the metallic state in the $\uparrow$ spin channel at *E*_*F*_ is dominated by Pd-${d}_{xy,yz}$ and Cl-${p}_{z}$ orbitals. The VBM of the $\downarrow$ spin channel originates from Pd-(${d}_{xz,yz}$/${d}_{{z}^{2},{x}^{2}{y}^{2}}$) and Cl-${p}_{y}$ states, while the CBM is derived from Pd-(${d}_{xz,yz}$/${d}_{{x}^{2}{y}^{2}}$) and Cl-${p}_{z}$ orbital states (see Fig. 1(c)). The intensity map of the electronic band structure is shown in Fig. 1(d).

**Figure 2:**
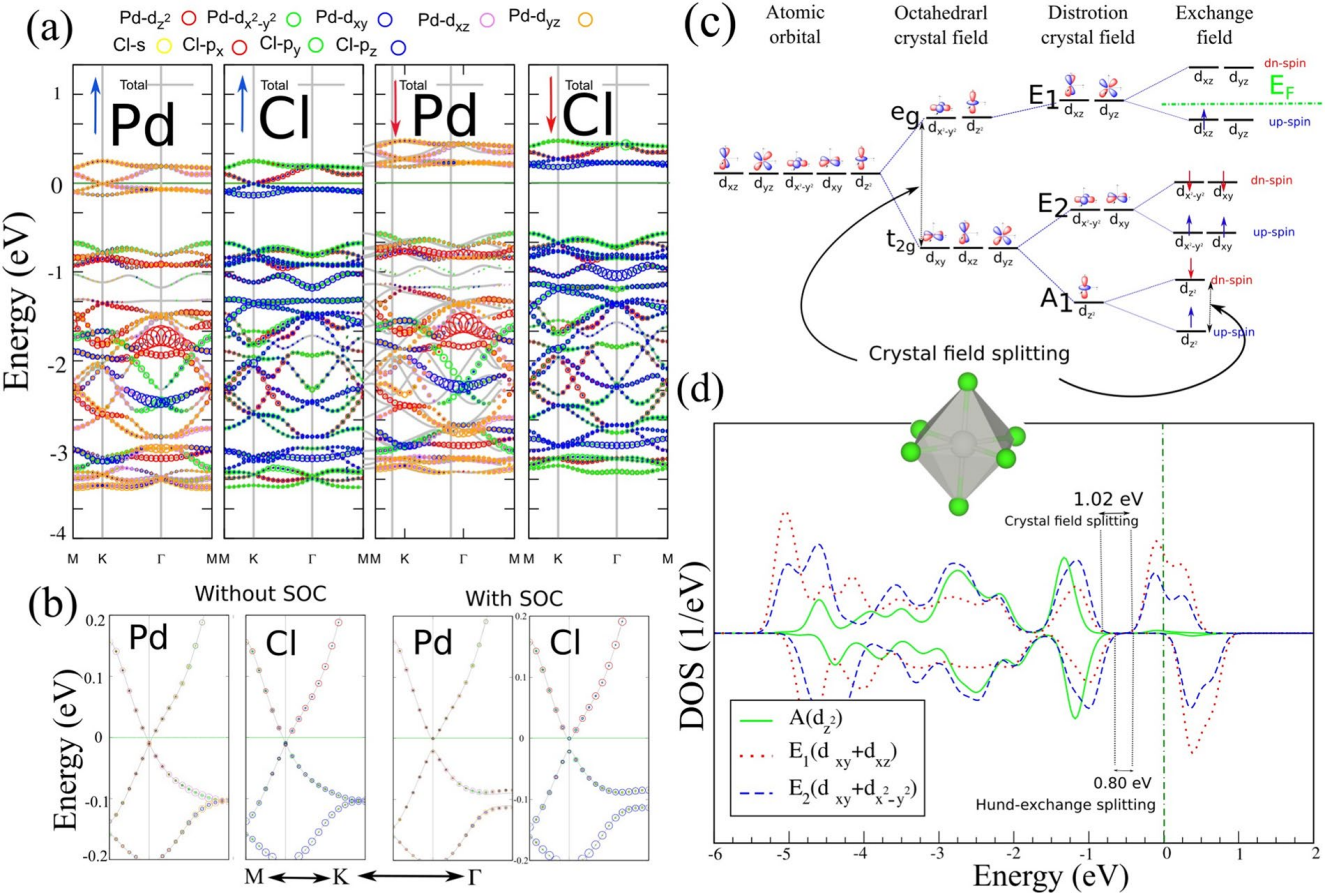
Orbital-projected electronic band structure of PdCl_3_ (**a**) without SOC and (**b**) on an enlarged scale with and without SOC (for the spin-up channel), (**c**) The schematic of crystal field and exchange splitting for Pd ions. (**d**) The PDOS of $d$ orbital states for Pd atoms is shown. The difference spin density distribution is shown as the inset. The blue and yellow regions represent the $\uparrow$ and $\downarrow$ spin states. The zero of energy is set at ${E}_{F}$.

Dirac states in Dirac materials, including the IV-based 2D honeycomb lattice, are characterized by being composed of $p$-orbital states with weak SOC. While the Dirac states of PdCl_3_ mainly originate from the Pd-*d* orbitals and upon the inclusion of SOC, despite the fact that Dirac-cone feature is preserved, a nontrivial energy band gap opens in the $\uparrow$ spin channel and TRS in the edge states is broken. The orbital-projected electronic band structure of PdCl_3_, on Pd and Cl atoms without spin-orbital coupling (SOC) and with SOC, are shown in Fig. 2(a,b). The states originate from ${E}_{2}$ (${d}_{xy}$, ${d}_{{x}^{2}{y}^{2}}$) with only a small contribution from the ${E}_{1}$ (${d}_{xz}$, ${d}_{yz}$) orbitals. Both the VB and CB exhibit equivalent weights of ${E}_{1}$ and ${E}_{2}$ orbital states, in calculations without SOC. With SOC, there are significantly increased contributions of both ${E}_{1}$ and ${E}_{2}$ in the CB while the ${E}_{1}$ and ${E}_{2}$ contributions to the VB decrease, resulting in the degeneracy of the ${E}_{1}$ and ${E}_{2}$ orbitals around the Dirac-point being lifted, opening thus a global energy gap between the CB and VB energies. Consequently, the ${E}_{1}$ and ${E}_{2}$ orbitals play a prominent role in the electronic topological properties of PdCl_3_. The larger SOC gap of Pd-*d* orbitals with broken TRS may lead to the Chern insulator and the QAH effect. The band gap is calculated to be 20 meV (with SOC) and 24 meV (with SOC + U) which is sufficiently large for the QAH effect to be observable.

To understand the origin of the magnetic moment in PdCl_3_, we investigate the crystal symmetry of the Pd-4*d* orbital states (see Fig. 2(c)). In PdCl_3_, each Pd atom is coordinated by six Cl atoms, forming a distorted octahedral crystal field. Also the Pd-4*d* orbital states split into three ${t}_{2g}$ (${d}_{xy,{x}^{2}{y}^{2}}$) and two ${e}_{g}$ (${d}_{xz,yz}$) orbital sub-states in a perfect octahedral crystal field. Due to the ${D}_{3d}$ point-group symmetry, structural distortion of the PdCl_6_ octahedron makes the ${t}_{2g}$ state further split into the ${A}_{1}$ (${d}_{{z}^{2}}$) and doubly-degenerate ${E}_{2}$ states, while they are still energetically lower than that of the ${E}_{1}$ states. Under an exchange field, **E**_*ex*_, as introduced by internal magnetism, the degeneracy of the ${A}_{1}$, ${E}_{2}$ and ${E}_{1}$ orbital states split significantly due to Hunds coupling. For a Pd^+3^ state, six electrons with both $\uparrow$ and $\downarrow$ spin channels fully occupy the ${A}_{1}$ and ${E}_{2}$ states, while only one electron is left in the $\uparrow$ spin channel of ${E}_{1}$, resulting in an integer magnetic moment.

To gain insight into the origin of its electronic, magnetic and topological properties, the PDOS of PdCl_3_ is shown in Fig. 2(d). We see that the Pd-*d* orbital states would be split into ${E}_{1}$, ${E}_{2}$ and ${A}_{1}$ states under the distorted octahedral crystal field of Cl atoms. In an octahedral crystal field PdCl_3_, based on Griffiths crystal field theory, the spin states of the TM ions can be determined by the relative strength between crystal field splitting (Δ${E}_{cf}$) and Hund-exchange splitting (Δ${E}_{ex}$) of Pd-*d* orbitals. The relative strength of the crystal field splitting is calculated Δ*E*_*cf*_ = 1.02 eV, that is, larger than Δ*E*_*ex*_ = 0.8 eV, resulting in a low spin (1 *μ*_*B*_) state which is in agreement with the Pd (${d}^{\uparrow 4}\downarrow 3$) spin configuration. The states around ${E}_{F}$ mainly originate from ${E}_{1}$ and ${E}_{2}$ orbitals, while the ${A}_{1}$ orbital does not contribute significantly. The partially occupied ${E}_{1}$ and ${E}_{2}$ orbitals around the ${E}_{F}$ form a Dirac-point in PdCl_3_.

### Magnetic order

In the following, we study the lowest energy structures of PdCl_3_ for different spin orientations, including nonmagnetic (NM), ferromagnetic (FM) and anti-ferromagnetic AFM) order. The electronic band structure of NM, FM and AFM orders are shown in Fig. S1(a–c). Clearly, the NM order exhibits a zero band gap due to the lowest ${t}_{2g}$ orbitals being half-filled with a 4*d*^8^ electronic configuration for the Pd^+3^ ions, leading to a metallic state. We find a Dirac-point in the $\uparrow$ spin channel of the FM state, up to ${E}_{F}$. In the case of AFM order, we find that the states shift to higher energy and peaks sharpen as compared to the FM order, and the system exhibits metallic character. In the AFM order, it can be seen that the $\uparrow$ and $\downarrow$ spin channels around ${E}_{F}$ almost overlap and do not show a Dirac-point. In the case of the FM state the magnetic moment is 2 *μ*_*B*_ per unit cell. The semiconducting energy band gap in the $\downarrow$ spin channel is 1.63 eV. A comparison of total energies for the ferromagnetic (FM), non-magnetic (NM), and antiferromagnetic (AFM) configurations show the lowest energy configuration to be FM for PdCl_3_. These results indicate that the magnetic ordering has important implications on the electronic band structures, especially at the band edges.

### Hubbard U

Due to the weak screening of the Coulomb interaction in 2DM, it is expected that the Hubbard U will be larger than in three-dimensional materials, thus the energy band gap may be expected to be enhanced significantly. We investigate effects of correlation by varying the value of the Hubbard U. The electronic band structure and PDOS of PdCl_3_ calculated for various values of *U* are shown in Figs. S2 and S3. The DOS of PdCl_3_ without SOC, as a function of the Hubbard U, is shown in Fig. S2(a) and the PDOS of the Pd atom *d* states is shown in Fig. S2(b). The correlation effects on the electronic and magnetic properties of PdCl_3_ are significant and cause changes in the spin polarization of PdCl_3_. We see from the PDOS that the PdCl_3_ structure still maintains the DHM feature even when considering the effect of the Hubbard *U*, and the magnetic moment is essentially unchanged (1.99 *μ*_*B*_ for U = 0.5 eV and is 2 *μ*_*B*_ for U = 4 eV). With increase of Hubbard U, the ${d}_{xz}$ and ${d}_{yz}$ orbital states do not change near ${E}_{F}$, indicating the robustness of nontrivial topology against the correlation effect in Pd-4*d* electrons. The electronic band structure of PdCl_3_ with SOC as a function of Hubbard U, is shown in Fig. S3(b). The band gap opening is a result of a cooperative effect of electron correlation with SOC. Our results show that the band gap of PdCl_3_ increases from 20 meV for U = 0.5 eV to 45 meV for U = 4 eV, and therefore QAHE remains for U = 4 eV. This trend is similar to that of ref. ^67^ which reported that the band gap varies from around 25 meV to 68 meV for values of U from 1.0 to 4.0 eV. This study furthermore reported a band gap of 63 meV as obtained from DFT calculations with the HSE06 hybrid functional.

## Layer thickness

**Figure 3:**
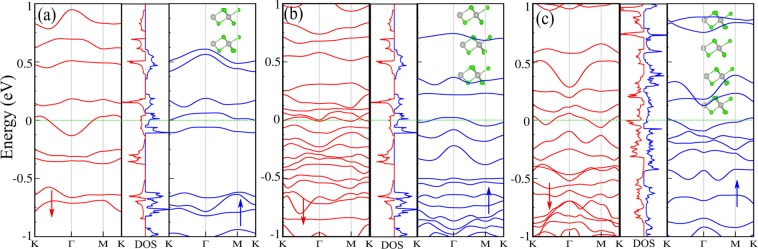
Optimized structures, electronic band structure and DOS of (**a**) 2L-PdCl_3_, (**b**) 3L-PdCl_3_ and (**c**) 4L-PdCl_3_. The zero of energy is set at ${E}_{F}$.

In order to understand the effect of layer thickness on the electronic properties, we perform band structure calculations for different thicknesses of PdCl_3_ (2L, 3L and 4L). The optimized structures and electronic band structure of 2L to 4L-PdCl_3_, are illustrated in Fig. 3(a–c), respectively. After geometry optimization and energy minimization, the interlayer distances between the layers of PdCl_3_ in the sandwich structures were obtained in the range of 2.548–2.582 Å. Moreover, for a few layer PdCl_3_, the bond length of Pd-Pd is in the range of 3.790–3.844 and Pd-Cl 2.458–2.533 Å. We find that the bond lengths increase compared to that of the PdCl_3_ monolayer (bond lengths of Pd-Pd and Pd-Cl in 1L-PdCl_3_ are 3.811 and 2.480 Å, respectively). Our results show that more than one layer of PdCl_3_, the electronic states are modified. In particular, 2L-PdCl_3_, exhibits half-metal behavior, namely the $\uparrow$ spin channel is an indirect semiconductor with a small band gap of 70 meV, where the touches ${E}_{F}$ and becomes gapless. The VBM and CBM of the $\uparrow$ spin channel are located at the $\Gamma$ and K points, respectively. The 2L-PdCl_3_ has 0.6 *μ*_*B*_ magnetic moment. We can see that the 3L-PdCl_3_ becomes a ferromagnetic-metal and has magnetic moment of 11 *μ*_*B*_ in the ground state;. 4L-PdCl_3_ also exhibits metallic character with a magnetic moment of 7 *μ*_*B*_. Thus, as the number of layers increases from 1L to 4L for the PdCl_3_ structure, the electronic states indicate a transition from a Dirac half-metal (1L) to half-metal (2L), and to a ferromagnetic-metal (3L and 4L), whree the calculated thickness are: 1L (3.590), 2L (8.124), 3L (13.492 Å) and 4L (18.518 Å). The thickness of 2L-PdCl_3_ (~8.124 Å) appears to be the critical thickness for such a DHM to metal transition. The nature of the band gap change in PdCl_3_ can be explained by a combination of quantum confinement and vdW interlayer interactions.

## Electric Field Effect On Bilayer PdCl_3_

**Figure 4:**
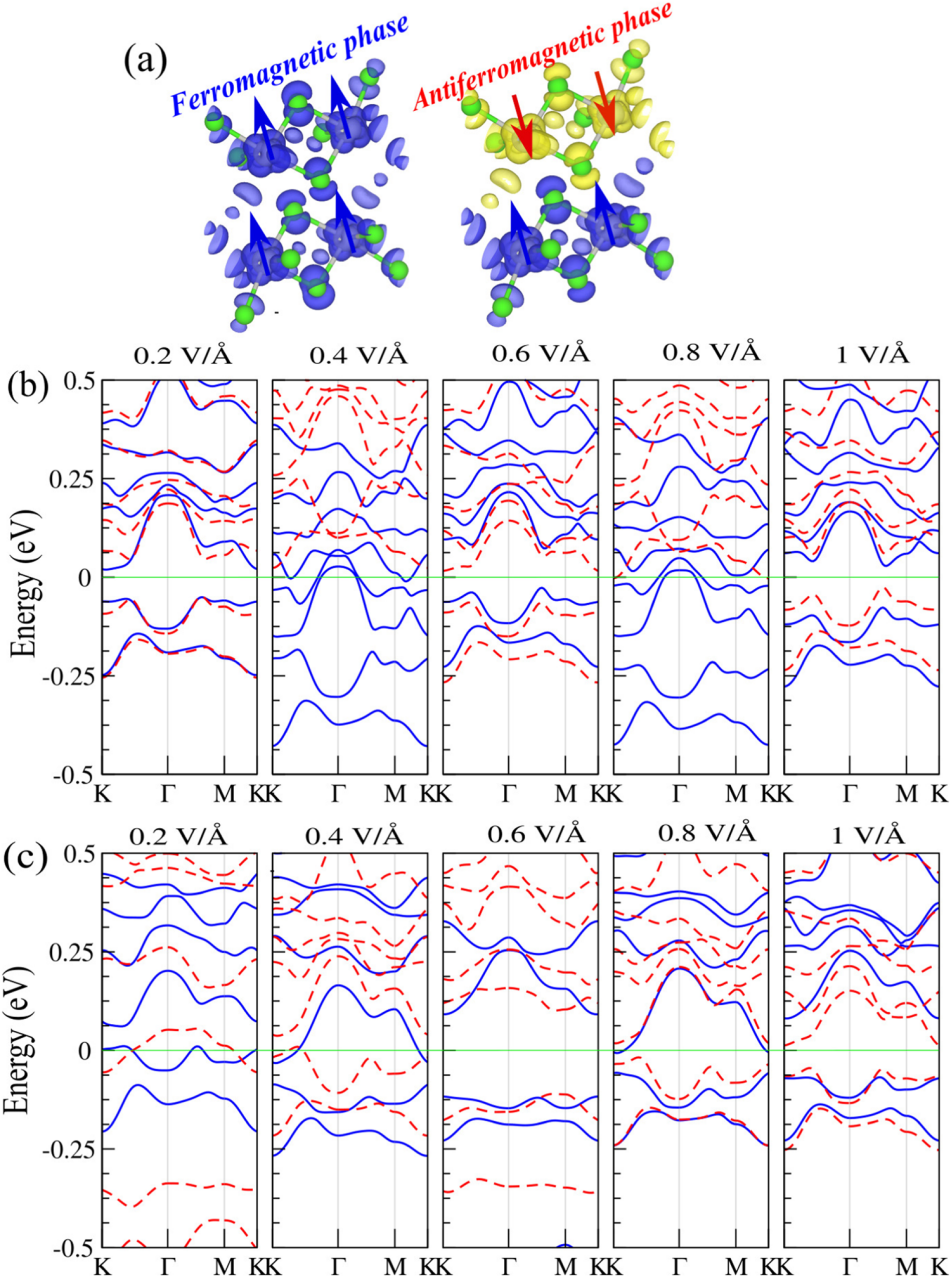
(**a**) Difference spin densities for the FM and AFM. The blue and yellow regions represent the $\uparrow$ and $\downarrow$ spin states, respectively. Electronic band structure of the PdCl_3_ bilayer in the (**b**) FM and (**c**) AFM configurations for different values of the electric field. The zero of energy is set at ${E}_{F}$.

In the following, we present the effect of an external uniform electric field on the electronic and magnetic properties of bilayer PdCl_3_ for two different spin configurations, namely, FM and AFM. The electric field is applied perpendicular to the plane of the PdCl_3_ bilayer. The schematic structure of the PdCl_3_ bilayer with two spin configurations under an electric field, is shown in Fig. 4(a). The ${{\rm{F}}}_{field} > 0$ denotes parallel to the z-axis and the value of electric field is considered between 0.2 and 1 V/Å. The band structures of PdCl_3_ bilayer in the FM and AFM configurations are shown in Fig. 4(b,c), respectively.

For the FM configuration, we found when the electric field increases from 0.2 to 1 V/Å, different electronic behaviour occurs including; dilute-magnetic semicounductor at 0.2, 0.6 and 1 V/Å with magnetic moment of 0.4, 0.35 and 0.2 *μ*_*B*_; half-metal (at 0.4 V/Å) and ferromagnetic-metal (at 0.8 V/Å) with 3.9 *μ*_*B*_ and 3.7 *μ*_*B*_ magnetic moments, respectively. We can see that the modification in the band structure occurs in both $\uparrow$ and $\downarrow$ spin channels in the AFM configuration, and leads to a transition to ferromagnetic-metal at 0.2 and 0.4 V/Å with magnetic moments of 0.9 and 0.4 *μ*_*B*_. While with incresing electic field to 0.6 V/Å and 1 V/Å, the PdCl_3_ bilayer becomes a dilute-magnetic semiconductor. The half-metallicity can be seen at 0.8 V/Å with 3.8 *μ*_*B*_ magnetic moment. As a result, it is possible to tune the electronic properties of PdCl_3_ bilayer and control the *E*_*F*_ by applying an electric field. The existence of the layered structure in the PdCl_3_, gives rise to a potential difference between the two atomic sublayers, which turns out to provide an useful tuning of the electronic and magnetic properties by a perpendicular electric field. Owing to their tunable band gaps and magnetism over a wide range, the layered PdCl_3_ material may have tremendous opportunities for application in nanoscale electronic and optoelectronic devices.

## Strain Effect

**Figure 5:**
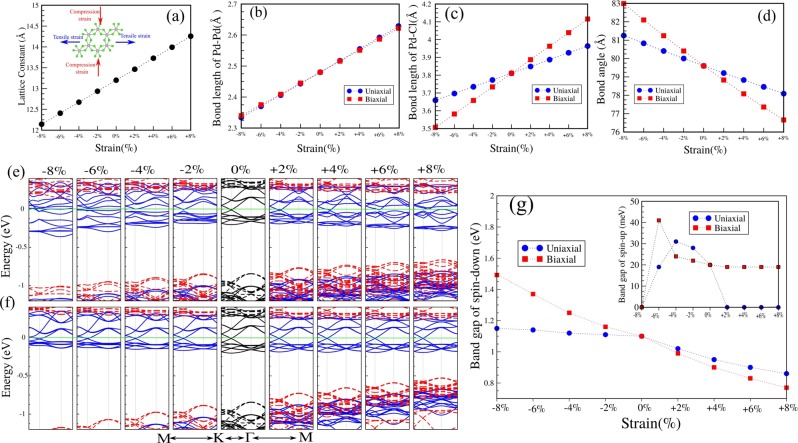
(**a**) Lattice constant and (**b**–**d**) bond lengths and bond angles as function of strain. The schematic structure of PdCl_3_ under in-plane uniaxial and biaxial strain for both tensile and compressive strain is shown in the inset of (**a**). (**e**,**f**) Electronic band structures of PdCl_3_ as function of uniaxial and biaxial strains. (**g**) Variation of the band gap as function of uniaxial and biaxial strain for PdCl_3_. The zero of energy is set at ${E}_{F}$.

Strain engineering is an effective approach to tune the electronic properties and the topological nature of 2DM. Here, we investigate the effects of uniaxial and biaxial strain on PdCl_3_. The schematic structure of PdCl_3_ under uniaxial and biaxial strain, is shown in Fig. 5(a). Maintaining the crystal symmetry, the tensile and compression strain are defined as $\varepsilon =(\tfrac{a-{a}_{0}}{{a}_{0}})\times 100$, where $a$ and ${a}_{0}$ are strained and non-strained lattice constants, respectively. The positive and negative values denotes tensile and compression states, respectively. Uniaxial strain is applied along the zigzag directions, while biaxial strain is applied along the *a*-*b* axis. The variation of the structural parameters, including the Pd-Pd and Pd-Cl bond lengths, bond angles and lattice constant as function of strain, are shown in Fig. 5(b–e). We find that the bond lengths and lattice constant, under both uniaxial and biaxial tensile (compression) strain from 0 to +8% (to −8%) increase (decrease) monotonically as expected. The electronic band structure of PdCl_3_ under uniaxial and biaxial strains are obtained as shown in Fig. 5(e,f). Notice that the value of the nontrivial band gap is modified significantly and therefore the topological nature of PdCl_3_ can be tuned by applying uniaxial or biaxial strain. Variation of band gap in the $\downarrow$ spin channel of PdCl_3_ as a function of uniaxial and biaxial strains, are shown in Fig. 5(h). For the $\downarrow$ spin channel of PdCl_3_, the band gap decreases monotonically as uniaxial and biaxial tensile strains are increased. This situation is different for compressive strain. Under a compressive biaxial strain, from 0% to −8%, the band gap increases. In the case of the $\uparrow$ spin channel (inset), the band gap is approximately invariant under biaxial strain from 0 to +8%, while from 0 to −6%, the band gap increases and becomes zero at −8%. For large uniaxial strain (>−6%), a DHM to HM transition is predicted to occur, while for biaxial compressive strain (>−8%), a DHM to dilute-magnetic semiconductor transition occurs. In contrast, under applied biaxial strain, the nontrivial topological states of PdCl_3_ are preserved.

## Vacancy Defects

**Figure 6:**
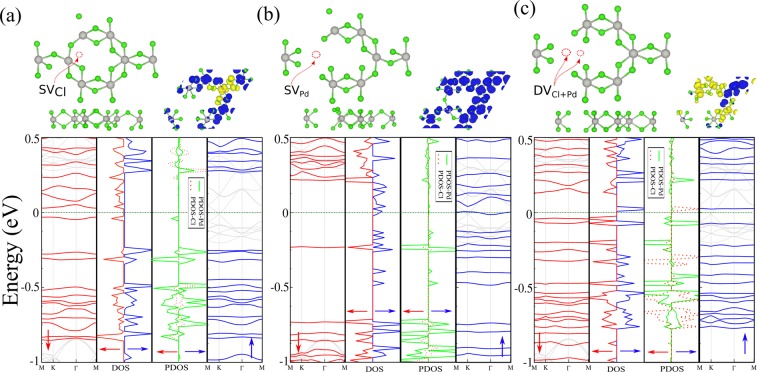
Optimized structures and electronic band structure with corresponding DOS and PDOS of PdCl_3_ with (**a**) a single Pd vacancy, (**b**) a single Cl vacancy and (**c**) the Pd + Cl double vacancy. The difference spin density distributions are shown in the insets. The blue and yellow regions represent the $\uparrow$ and $\downarrow$ spin states, respectively. The zero of energy is set at ${E}_{F}$.

In this section, we study effects of topological defects (including single and double vacancies) on the structural, electronic and magnetic properties of PdCl_3_. We remove Pd and Cl atoms to produce single vacancies of the Pd atom ($S{V}_{Pd}$) or Cl atom ($S{V}_{Cl}$), while for double vacancies, we remove both the Pd + Cl ($D{V}_{PdCl}$) atoms. The defective structures of PdCl_3_ are fully relaxed and accurate ground state energies and band structures are obtained. The optimized atomic structures of PdCl_3_ with vacancy defects are shown in Fig. 6. The bond length between Pd-Cl and Pd-Pd atoms in the vacancy structures is ~2.343 and 3.686 Å, respectively. We find that compared to pristine PdCl_3_ (Pd-Pd and Pd-Cl bond lengths are 3.811 and 2.480 Å) the bond lengths decrease around the vacancy defects. In order to better identify and investigate the effects of defects on PdCl_3_, difference charge densities and simulated STM images for different vacancies of PdCl_3_, are shown in Fig. S4. We see that the Cl atoms appear as white spots, however, the region around the vacancy defects corresponds to the brightest spot.

By performing spin-polarized calculations, we calculate the electronic and magnetic properties of PdCl_3_ in the presence of vacancy defects as shown in Fig. 6(a–c). We see that the $S{V}_{Cl}$ exhibits a dilute-magnetic semiconductor character with direct band gaps of 75 meV and 0.34 eV in the $\uparrow$ and $\downarrow$ spin channels, respectively (the VBM and CBM are located at the M point in both spin channels) and has a magnetic moment of 2 *μ*_*B*_. It is also seen from the DOS and PDOS that the state at ${E}_{F}$ is due to the missing Pd atom, which confirms the semiconducting behavior of $S{V}_{Pd}$ and also the magnetism is derived from these states. Unlike the $S{V}_{Cl}$, the $S{V}_{Pd}$ exhibits a half-metal character with a direct band gap of 0.72 eV, where the VBM and CBM are located at the $\Gamma$ and M points, in the $\downarrow$ spin channel. The $\uparrow$ spin channel presents a metallic character with the electronic bands crossing ${E}_{F}$. A magnetic moment of 6.21 *μ*_*B*_ is induced due to the dangling bonds around the single vacancy and an unpaired electron. From the DOS and PDOS, we see that the state at ${E}_{F}$ originates from orbitals around the Pd atom, which gives rise to the metallic behavior of the $S{V}_{Pd}$ in the $\uparrow$ spin channel. Similarly to $S{V}_{Cl}$, the $D{V}_{PdCl}$ defect exhibits a dilute-magnetic semiconductor character with a direct band gap of 0.67 eV where the VBM and CBM are located at the $\Gamma$ point, and there is an induced magnetic moment of 0.55 *μ*_*B*_. To obtain further insight into the character of the bonding, the difference electron charge density distributions of defective PdCl_3_ are presented in Fig. S7. We can see that there is an obvious charge accumulation around the Cl vacancies. The difference in spin densities are presented in the same panel in Fig. 6(a–c).

## Adsorption of Adatoms

In the following we investigate the adsorption of alkali metal (AM) including Li, Na, K and alkali earth metal (AEM) and Be, Mg, Ca and Al adatoms on PdCl_3_. The optimized structures, with corresponding difference charge densities, are shown in Fig. S5(a,b). The Li, Na, K, Mg, Ca and Al adatoms bond to the six Cl atoms of PdCl_3_ in the stable H-site and do not show any significant distortion. The *d*_*A*–*Cl*_ of these adatoms is in the range of 2.314–3.029 Å. Of the adatoms, Be binds most strongly to PdCl_3_, and has the shortest bond length, *d*_*A*–*Cl*_ = 2.314 Å, and the largest for K with *d*_*A*–*Cl*_ = 3.029 Å. The height of the adatoms above the surface is calculated as the difference between the average coordinates of neighboring Cl atoms and the adatom, and is in the range of 1 Å. We see that the *d*_*Pd*–*Cl*_ bond lengths in the Ad/PdCl_3_ structures, are in the range of 2.516–2.697 Å, which compared to those in PdCl_3_ (Pd-Pd and Pd-Cl are 3.811 and 2.48 Å) the bond length of Pd-Cl is increased. These adatoms do not have a significant effect on altering the positions of the neighboring Cl atoms, and the deformation in the PdCl_3_ structure is negligible.

We calculated the adsorption energy is defined as ${E}_{a}={E}_{Ad/PdC{l}_{3}}$-${E}_{PdC{l}_{3}}$-${E}_{Ad}$, where ${E}_{Ad/PdC{l}_{3}}$ is the total energy of the structure with adatom adsorption on PdCl_3_, ${E}_{PdC{l}_{3}}$ is total energy of pristine PdCl_3_ without adatoms, and ${E}_{Ad}$ is the total energy of an isolated adatom in vacuum. We labeled adatom adsorbed on PdCl_3_ as Ad/PdCl_3_. The determined adsorption energies of Li, Na, K, Be, Mg Ca and Al atoms adsorbed on PdCl_3_ are 3.50, 2.75, 2.49, 4.10, 4.39, 5.12 and 5.51 eV, respectively. These values are all larger than the respective (experimental) cohesive energies (see Table S1 of the SI), indicating that formation of these structures is favourable compared to forming clusters on the surface. The difference charge densities of Ad/PdCl_3_ are shown in Fig. S5(b). We see that strong electron depletion occurs on the adatoms, while there is a local electron gain on the Cl hexagon, which demonstrates the largely ionic character of the Ad-Cl bonds.

**Figure 7:**
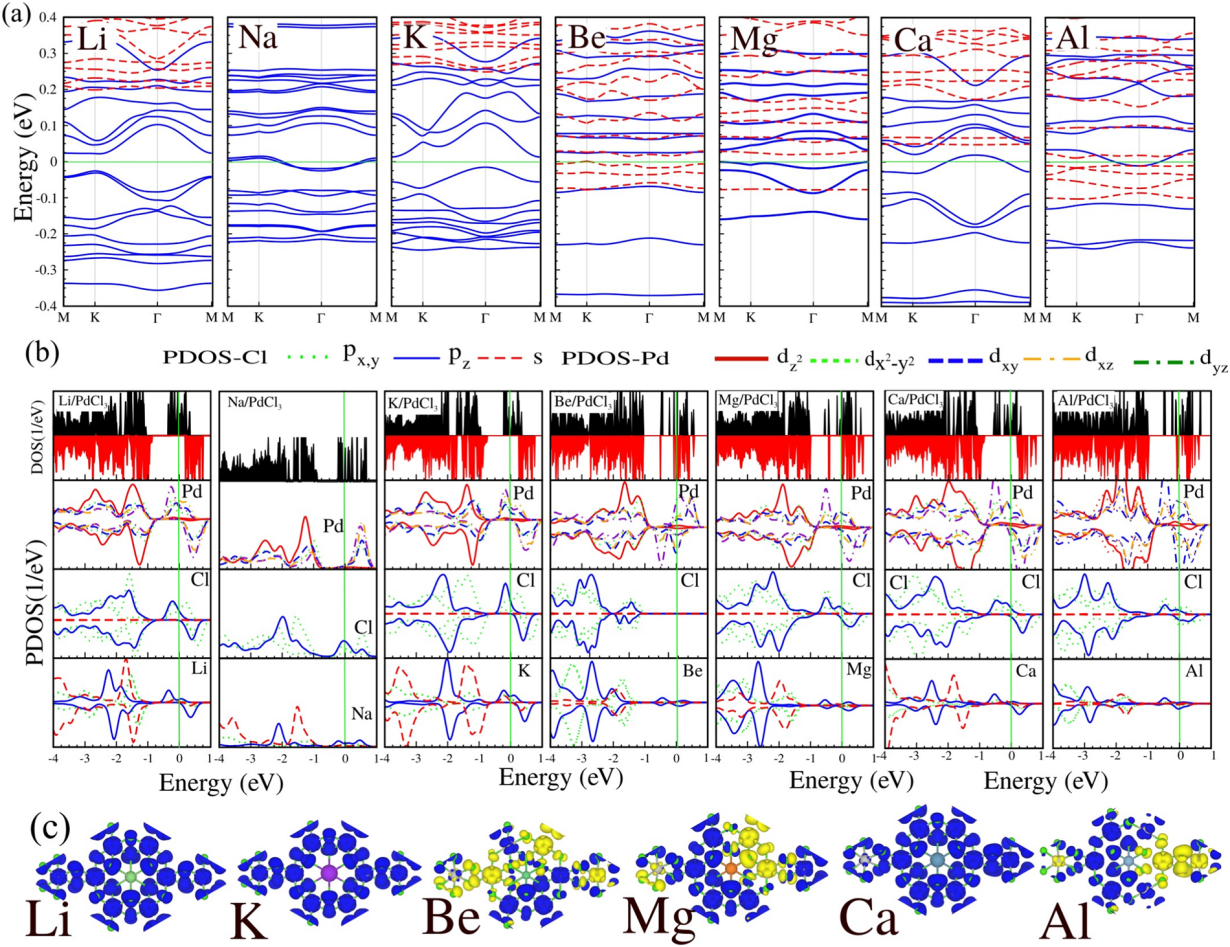
(**a**) Electronic band structure and (**b**) corresponding DOS and PDOS and (**c**) difference spin density of adsorbates on PdCl_3_: Li, Na, K, Be, Mg, Ca and Al adatoms. The blue and yellow regions show the $\uparrow$ and $\downarrow$ spin states, respectively. The zero of energy is set at ${E}_{F}$.

The electronic band structures, corresponding DOS/PDOS of Ad/PdCl_3_ (Ad = Li, Na, K, Be, Mg, Ca and Al), and difference spin densities are given in Fig. 7(a–c). Compared to the band structure of pristine PdCl_3_, it can be seen that the adsorption bands of these adatoms mainly concentrate around ${E}_{F}$, which are formed by the hybridization between AM-*s* with Pd-$d$ and Cl-$p$ orbital states. We see from the band structure of Li and K/PdCl_3_, that the energy bands split into $\uparrow$ and $\downarrow$ spin channels, and so these adsorbate structures become dilute-magnetic semiconductor-like. After adsorption of Li and K atoms, the band structure exhibits a small direct band gap of respectively, 50 meV and 30 meV in the $\uparrow$ spin channel, while the $\downarrow$ spin channel has a band gap of 1 eV. The magnetic moment (per unit cell) for Li and K in the ground state is 9 *μ*_*B*_, while the Na/PdCl_3_ system becomes a nonmagnetic metal. From the DOS and PDOS, it can be seen that for Na the metallic character (sharp peaks) near ${E}_{F}$ originate from the Cl-$s$ and Na-$s$ orbitals. The Be/PdCl_3_ system becomes a half-metal with an indirect band gap of 0.1 eV, where the VBM and CBM are located at the M and $\Gamma$ points, respectively, and induces a magnetic moment of 0.72 *μ*_*B*_.

The adsorption of Mg leads to a structure that exhibits a spin-glass semiconductor character, namely, the $\uparrow$ spin channel is gapless, while the $\downarrow$ spin channel is a direct semiconductor with a band gap of 100 meV and the VBM of the $\downarrow$ spin channel touches the Fermi level. With adsorption of Mg, the degeneracy of both spin channels are broken and a magnetic moment of 3.3 *μ*_*B*_ is induced. For Ca and Al adsorption, impurity states appear in the vicinity of ${E}_{F}$, and exhibit metallic characteristics in the $\uparrow$ spin channel, while in the down spin channel it exhibits semiconducting character with band gaps of 1 eV and 20 meV, respectively. The half-metallic character of the Ca and Al systems induces magnetic moments of 9.6 and 1.8 *μ*_*B*_. From the DOS and PDOS, shown in Fig. 7(b), we see that the state at ${E}_{F}$ has Ca/Al-$s$ orbital character which confirms the metallic behavior in the $\uparrow$ spin channel. It can also be seen from the DOS and PDOS shown in Fig. 7(b), that the VBM of the Mg and Ca structures consists of $s$ and ${p}_{y}$-orbitals and the CBM consists of ${p}_{x,z}$-orbitals, which confirms the semiconducting behavior of these structures. The difference spin density that is calculated from the charge density difference between the $\uparrow$ and $\downarrow$ spin channels, are shown in Fig. 7(c).

**Figure 8:**
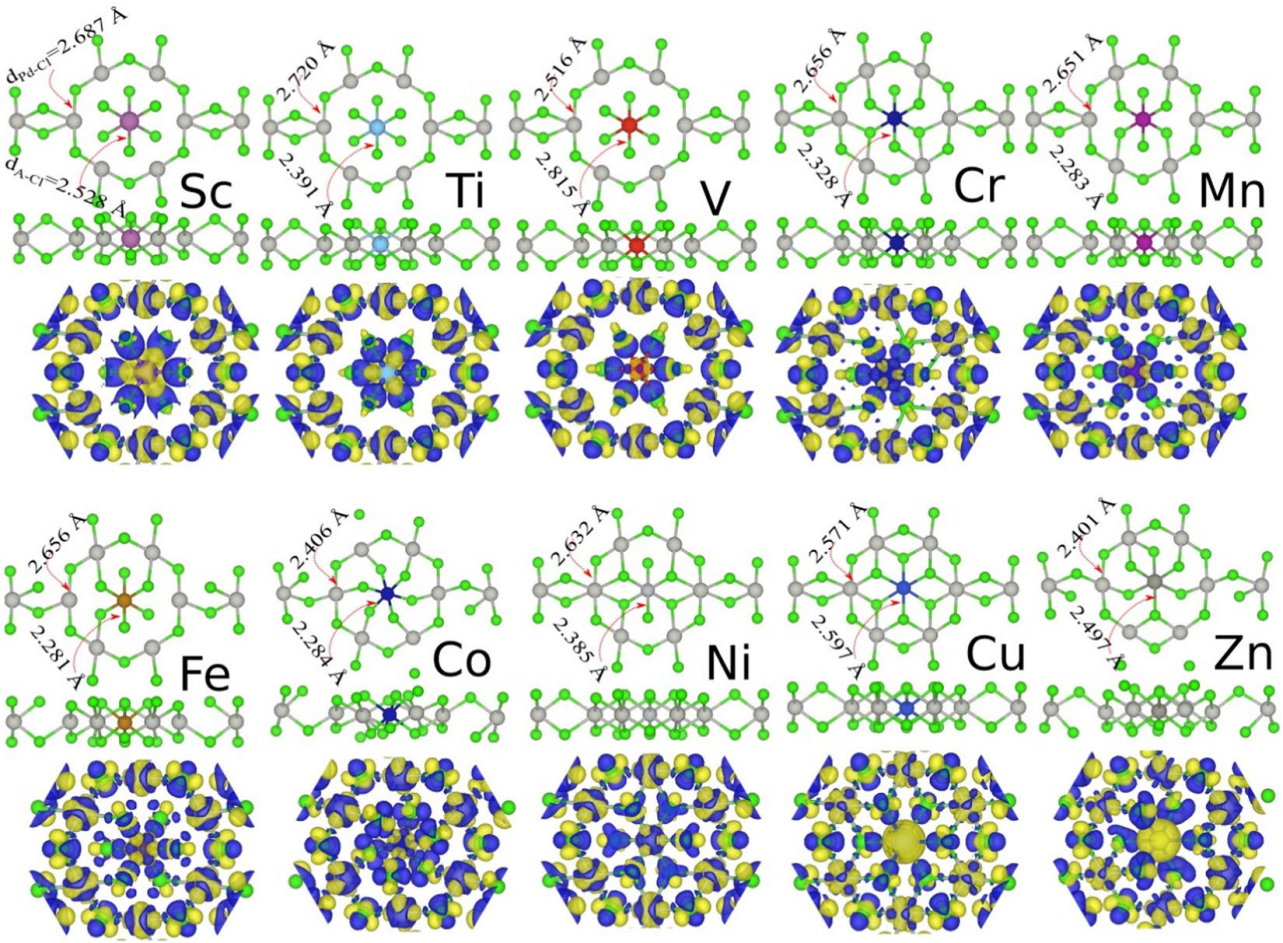
Optimized atomic structures and the difference charge densities of adatoms adsorbed on PdCl_3_, namely, Sc, Ti, V, Cr, Mn, Fe, Co, Ni and Zn adatoms. The blue and yellow regions represent the charge accumulation and depletion, respectively.

The relaxed structures of the TMs/PdCl_3_ systems (TM = Sc, Ti, V, Cr, Mn, Fe, Co, Ni, Cu and Zn adatoms) at the stable H-site, are shown in Fig. 8. We see that the TM adatoms, similarly to the AM, bind to the six Cl atoms of PdCl_3_. Unlike the AM and AEM adatoms however, which only weakly interact with the PdCl_3_ structure, adsorption energy of TMs adatoms on PdCl_3_, can result in a relatively stronger interaction between the TMs and PdCl_3_ and a smaller *d*_*Ad*–*Cl*_ distance. The bond length, *d*_*Ad*–*Cl*_, of the TM adatoms is in the range of 2.281 (Co)–2.528 (Sc)Å. The height of adatoms is calculated as the difference between the average coordinates of neighboring Cl atoms and the adatom and is in the range of 1 Å. The *d*_*Pd*–*Cl*_ bond length in the Ad/PdCl_3_ structure, is in the range of 2.401–2.720 Å, which in comparison to PdCl_3_ (2.480 Å) is greater. The adsorption of TMs adatoms can result in relatively stronger interaction with values of 4.92, 5.23, 6.54, 6.97, 7.28, 7.29, 6.81, 4.92, 3.90 and 2.53 eV, respectively. Similarly to the AM and AEM adatom systems discussed above, all the adsorption energies of the TM are larger than the respective cohesive energies indicating the metal atoms will prefer to be bonded in the nanosheet compared to forming metal clusters on the surface (see Table S1 of the SI). Of the TM adatoms, Co and Sc have the largest and smallest bond lengths, respectively. We see that some of the adatoms, such as Co and Zn, exhibit a shifting of the position of the neighboring Cl atoms, resulting in a noticeable deformation in the PdCl_3_ structure. The difference charge densities of TMs/PdCl_3_, are also shown in Fig. 8. Considering, for example Cr/PdCl_3_, a charge accumulation appears in the region of the Cr atom and the neighboring Cl atoms, exhibiting a strongly covalent bonding character in the formed *d*_*Cr*–*Cl*_ bond. Such stronger covalent bonding is also found in most of the other TMs/PdCl_3_.

**Figure 9:**
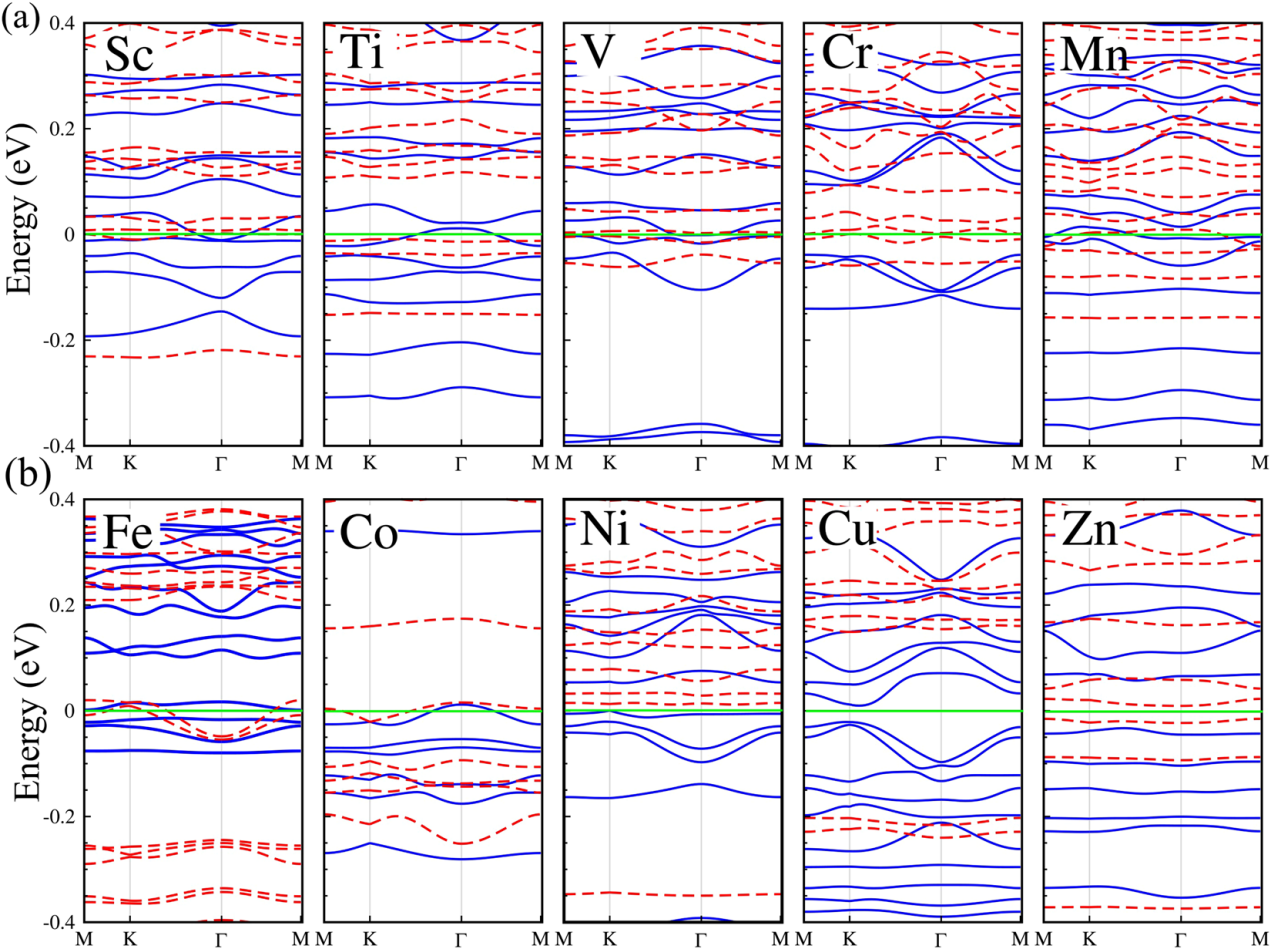
Electronic band structure of adsorbates on PdCl_3_; (**a**) Sc, Ti, V, Cr, Mn and (**b**) Fe, Co, Ni and Zn adatoms. The Fermi energy at zero eV is represented by the horizontal green line.

The electronic band structure of the TM/PdCl_3_ systems are shown in Fig. 9 and the DOS and PDOS in Fig. S6. The $d$- and 4$s$-orbitals of the TMs have similar energies, and the initial magnetic moment of the isolated TMs are nonzero unless the 3$d$-orbital shell is completely occupied. As the 3$d$-orbitals are located near to the nucleus, TMs with their partially occupied 3$d$-orbital shells can exhibit a diversity of electronic properties. The adsorbate’s Sc, Mn and Co, exhibit a ferromagnetic-metal character with induced magnetic moments of 4.55, 7.93 and 2.0 *μ*_*B*_, respectively. The metallic state originates from the $4s(\uparrow )$ and $3{d}_{{z}^{2}}(\downarrow )/3{d}_{xy}$ orbital states at ${E}_{F}$. With adsorption of Ti, V, Cr and Fe on PdCl_3_, the system remains metallic in the electron $\uparrow$ spin channel, whereas the $\downarrow$ spin channel bands exhibit semiconductor character, resulting in half-metal systems. The magnetic moments of Ti, V, Cr, Fe/PdCl_3_ are 7.14, 6.90, 9.71 and 4.33 *μ*_*B*_, respectively. The Ti- and V-adsorbate systems have 0.1 eV and 5 meV indirect band gaps in the $\downarrow$ spin channel, respectively. While the Cr- and Fe-adsorbate structures have indirect band gaps of 30 meV and 15 meV, respectively, in the $\uparrow$ spin channel.

From the band structure in Fig. 9(b), we see that the Ni/PdCl_3_ structure becomes a spin-glass semiconductor with 50 meV and 100 eV indirect band gaps in the $\uparrow$ and $\downarrow$ spin channels, respectively, with an induced magnetic moment of 8.63 *μ*_*B*_. The Cu/PdCl_3_ structure has band gaps of 1 eV and 30 meV in the $\uparrow$ and $\downarrow$ spin channels, respectively. This structure in the ground state has a large magnetic moment of 9.5 *μ*_*B*_. With adsorption of Zn on PdCl_3_, the structure becomes a dilute-magnetic semiconductor and induces a magnetic moment of 5.80 *μ*_*B*_. The spin density differences of the TMs/PdCl_3_ systems are shown in Fig. S7. The charge carriers around *E*_*F*_ are mobile, which is not only useful for conductive behavior but also for magnetic coupling. The difference spin density of Sc, Cu/PdCl_3_, show that the sign of spin density about the Sc and Cu adatoms and its six Cl atom neighbors are opposite, thus displaying a (weak) FM interaction between them. In other systems, we can observe highly localized opposite spin densities, thus indicating the presence of an AFM interaction in these doped structures.

## Edge States

**Figure 10:**
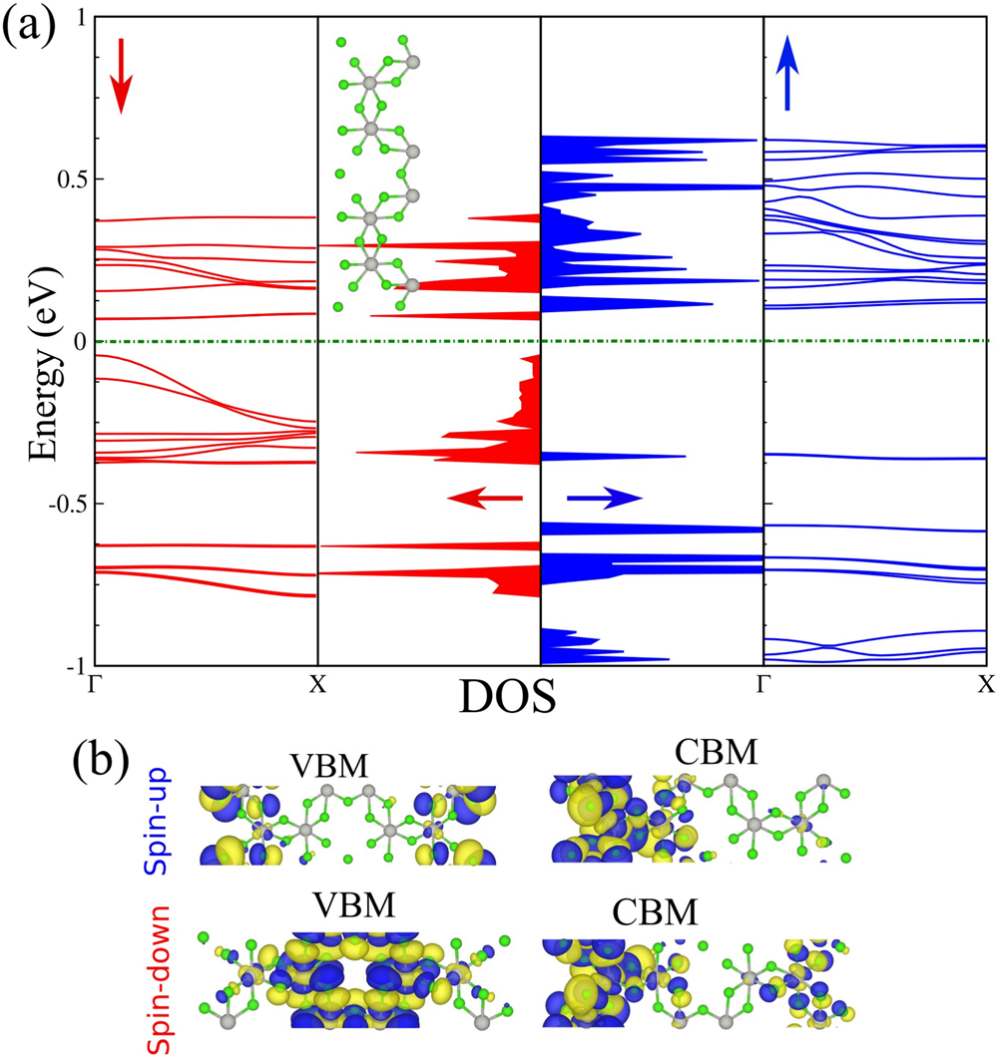
(**a**) Electronic structure and DOS of Z-PdCl_3_NR. The optimized atomic structure is shown in the inset. The zero of energy is set at ${E}_{F}$. (**b**) The VBM and CBM electronic states.

By cutting a PdCl_3_ nanosheet along the $x$- or $y$-directions we obtain a PdCl_3_ nanoribbon (PdCl_3_NR) with two types of edge configurations. Such ribbons exhibit a rich variation of electronic properties and characteristics due to its edge states, which depend on the width, which may provide important features for various technological applications. Two major families of PdCl_3_NRs are distinguished depending on their orientations, namely, armchair and zigzag PdCl_3_NRs. For the armchair edge states, the width, $n$ is defined by the number of Pd-Cl chains in the unit cell which are parallel to the axis of the nanoribbon, while for the zigzag edge states, $n$ denotes the number of zigzag Pd-Cl chains along the nanoribbon axis. For convenience, they are specified as $n$Z-PdCl_3_NR and PdCl_3_NR, respectively. In the present work, one type of PdCl_3_NR with zigzag edges is investigated. The schematic structure of the Z-PdCl_3_NR is show in Fig. 10 (inset). The electronic band structure and DOS of Z-PdCl_3_NR, is also shown in Fig. 10(a). The VBM or CBM are determined by edge states and quantum confinement effects, thus the electronic band structure is modified significantly as compare to the infinite sheet. The charge densities of the VBM and CBM are shown in Fig. 10(b). We find that the DHM characteristic of the PdCl_3_ nanosheet is transformed to that of a dilute-magnetic semiconductor with a magnetic moment of 6.0 *μ*_*B*_.

## Optical Properties

**Figure 11:**
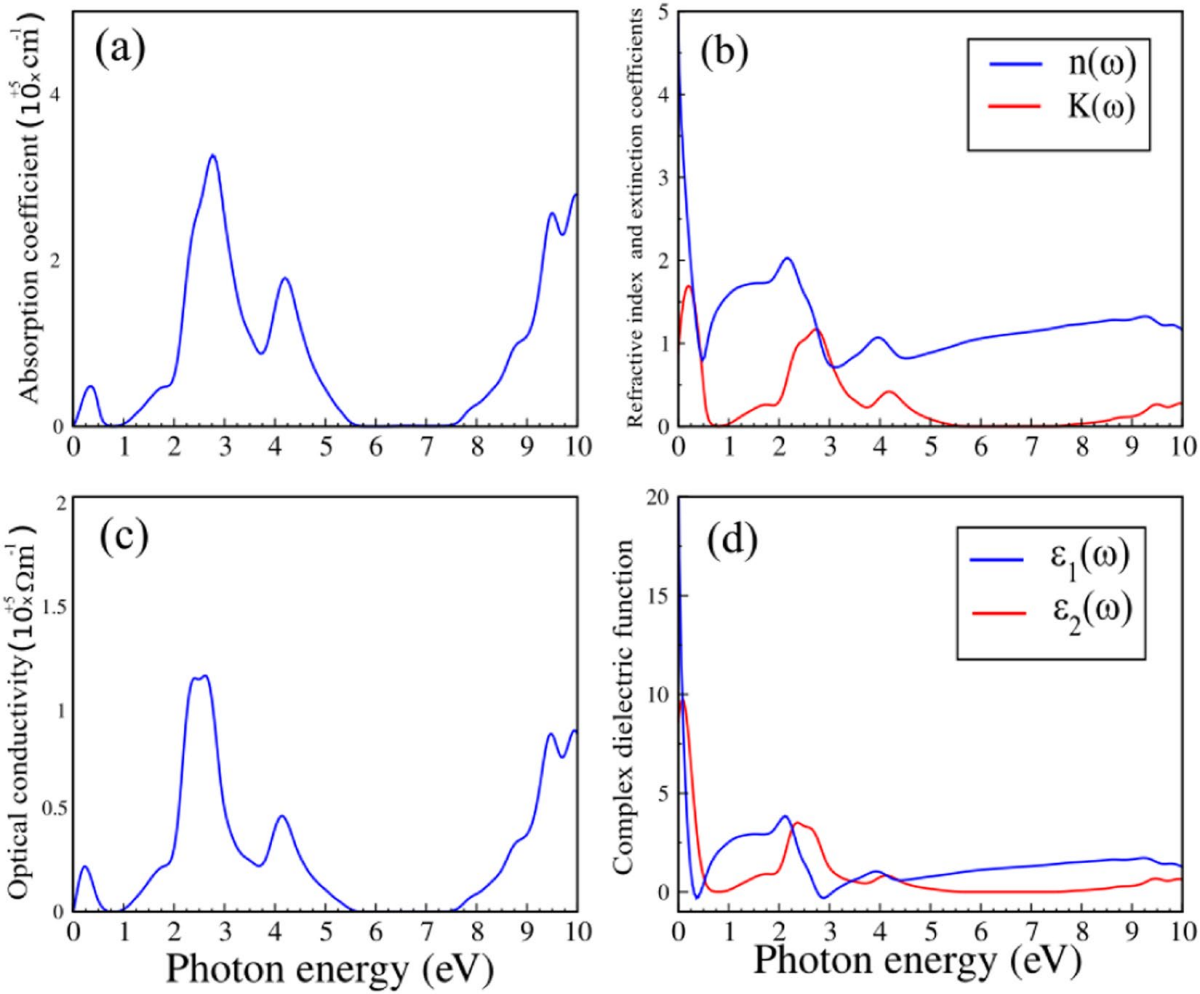
(**a**) Absorption coefficient, (**b**) refractive index and extinction coefficient, (**c**) the optical conductivity and (**d**) the real and imaginary parts of the complex dielectric function of PdCl_3_.

Since PdCl_3_ is a semiconductor in the $\downarrow$ spin channel, it is important to investigate its optical manifestations. Here, we compute the optical properties using SIESTA, which are constructed from the orbital atomic functions converged by the PBE potential. In particular, we calculated the optical properties: the dielectric function, absorption coefficient, reflectivity, refractive index ($n(\omega )$) and extinction coefficient ($K(\omega )$) of PdCl_3_. The absorption coefficient is a percentage that tells the decay of light intensity spreads in unit distance in the medium as shown in Fig. 11(a). We see that the absorption coefficient is almost zero when the energy is in the range of 0.62–1.02 eV, and there is no electronic transition because the energy of the photon is lower than the band gap of PdCl_3_ (1.12 eV). Also, in the range of 5.62–7.47 eV (ultraviolet region), the absorption coefficient is zero. When the photon energy is larger than the value of the band gap, the absorption coefficient will increase. The absorption spectrum starts from about 4.773 × 10^4^ at 0.34 eV, after which the intensity varies with increase in photon energy, and reaches a maximum value of 32.5 × 10^4^ at 2.78 eV. A secondary peak is located at the value of 17.85 × 10^4^ at 4.20 eV. The major peak appears in a broad energy range of 2.7–4.2 eV, which indicates the pronounced absorption of visible light (2.5–4.5 eV) in PdCl_3_. The refractive index and extinction coefficient, are presented in Fig. 11(b).

The static refractive index is calculate to be *n*(0) = 4.50 and the maximum frequency of $n(\omega )$ is 2 at 2.16 eV. At high photon energy, the refractive index eventually tends to the value of one, while increases and decrease with energy in both the IR and Visible region. From $K(\omega )$ the local maxima of the extinction coefficient corresponds to the zero of ${\varepsilon }_{1}(\omega )$ ~ 0.21 eV. The spectrum curve of $n(\omega )$ and $K(\omega )$ rapidly decreases with increasing photon energy in the UV region and it will becomes constant after 20 eV. The real (absorptive) parts of the optical conductivity of PdCl_3_ are shown in Fig. 11(c). We see that the peak absorption happens at the photon energy of 2.52 eV and reduces gradually to zero at the high energy region. The real (${\varepsilon }_{1}(\omega )$) and imaginary (${\varepsilon }_{2}(\omega )$) parts of the complex dielectric function, is shown in Fig. 11(d). The real part of the dielectric function gives information about the electronic polarizability of the material. We see that the static dielectric constant in the zero frequency limit is obtained as ${\varepsilon }_{1}(0)$ = 19. The quantity ${\varepsilon }_{1}(\omega )$ can become negative and this indicates that PdCl_3_ exhibits a metallic behavior in this frequency region. For ${\varepsilon }_{2}(\omega )$, there are mainly two peaks, which are 9.7 at 0.09 eV and 2.37 at 3.45 eV. In PdCl_3_, peaks in the real and imaginary part of the dielectric function are mainly due to electronic transitions from 4$d$-orbital in the valence band to 5$s$-orbital in the conduction band and there is also possible an electronic transition from 5$s$-orbital in the valence band to 5$p$-orbital in the conduction band. It is well known that materials with band gaps below 1.55 eV work well in the infrared (IR) and Visible region of the spectrum. Therefore PdCl_3_ will function in the IR and Visible region as an optical material.

## Conclusion

In summary, by using first-principles calculations, we have systematically investigated the effects of adatom adsorption, vacancy defects, electric field, strain engineering, edge states and layer thickness on the structural, electronic and magnetic properties of the 2D honeycomb metal-halogen lattice, PdCl_3_ (paladium trichloride) monolayer. Our calculations show that PdCl_3_ is a Dirac half-metal (DHM) and exhibits a QAHE with a large non-trivial band gap of ~25 meV. We found that PdCl_3_ prefers a ferromagnetic spin orientation in the plane for Pd atoms with a 2 *μ*_*B*_ magnetic moment. The effect of correlation in the Pd-4$d$ orbitals and SOC is investigated by including the on-site Coulomb repulsion U, We find that this results in an increase of the band gap (from 20 meV for *U* = 0.5 eV to 45 meV for *U* = 4 eV). Our results show that with increasing number of layers from monolayer to quadlayer, the electronic structure indicates a transition from Dirac half-metal (monolayer) to half-metal (bilayer) to a ferromagnetic metal (trilayer, quadlayer). Furthermore, when an electric field is applied perpendicular to the bilayer PdCl_3_, the band gap decreases, while under uniaxial and biaxial strain, the band structure can also be modified. With adsorption of adatoms, PdCl_3_ can be turned into a metal (Na), half-metal (Be, Ca, Al, Ti, V, Cr, Fe and Cu); ferromagnetic-metal (Sc, Mn and Co); spin-glass semiconductor (Mg, Ni); dilute-magnetic semiconductor (Li, K and Zn), while single (Pd, Cl) and divacancy defects (Pd + Cl) induce dilute semiconductor and metallic properties, respectively. Through these functionalization and bandstructure engineering approaches, the electronic properties and magnetism of PdCl_3_ nanosheets can be tuned.

## Supplementary information

Supplementary Information.
