# Supplementary material for: Dirac half-metallicity of Thin PdCl3 Nanosheets: Investigation of the Effects of External Fields, Surface Adsorption and Defect Engineering on the Electronic and Magnetic Properties
Source: Sci Rep. 2020 Jan 14;10:213. doi: 10.1038/s41598-019-57353-3 (PMC6959269; doi:10.1038/s41598-019-57353-3)
Supplement: Supplementary file 1 — Supplementary Information. [file 41598_2019_57353_MOESM1_ESM.pdf]

Supplementary Information - Dirac half-metallicity of Thin $PdCl_3$ Nanosheets: Investigation of the Effects of External Fields, Surface Adsorption and Defect Engineering on the Electronic and Magnetic Properties

Asadollah Bafekry*[1,2] , Catherine Stampfl[3] , & Francois M. Peeters[2]

[1]Department of Physics, University of Guilan, 41335-1914 Rasht, Iran
[2]Department of Physics, University of Antwerp, Groenenborgerlaan 171, B-2020 Antwerp, Belgium
[3]School of Physics, The University of Sydney, New South Wales 2006, Australia

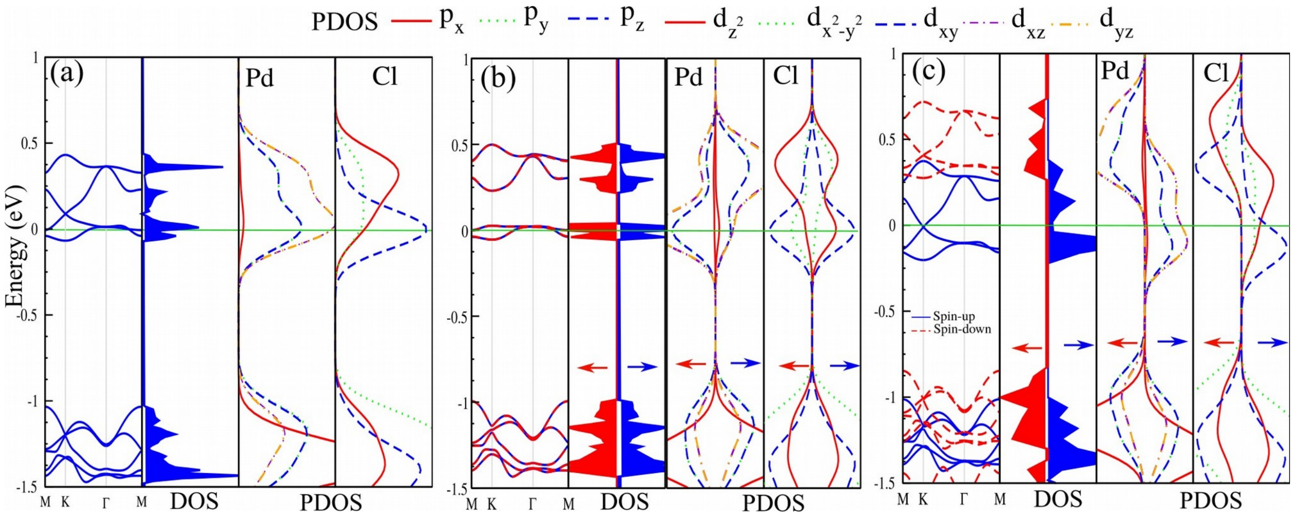

Figure. S1: Electronic band structure and PDOS of $PdCl_3$ for (a) nonmagnetic, (b) antiferromagnetic, and (c) ferromagnetic order. The zero of energy is set at E_F .

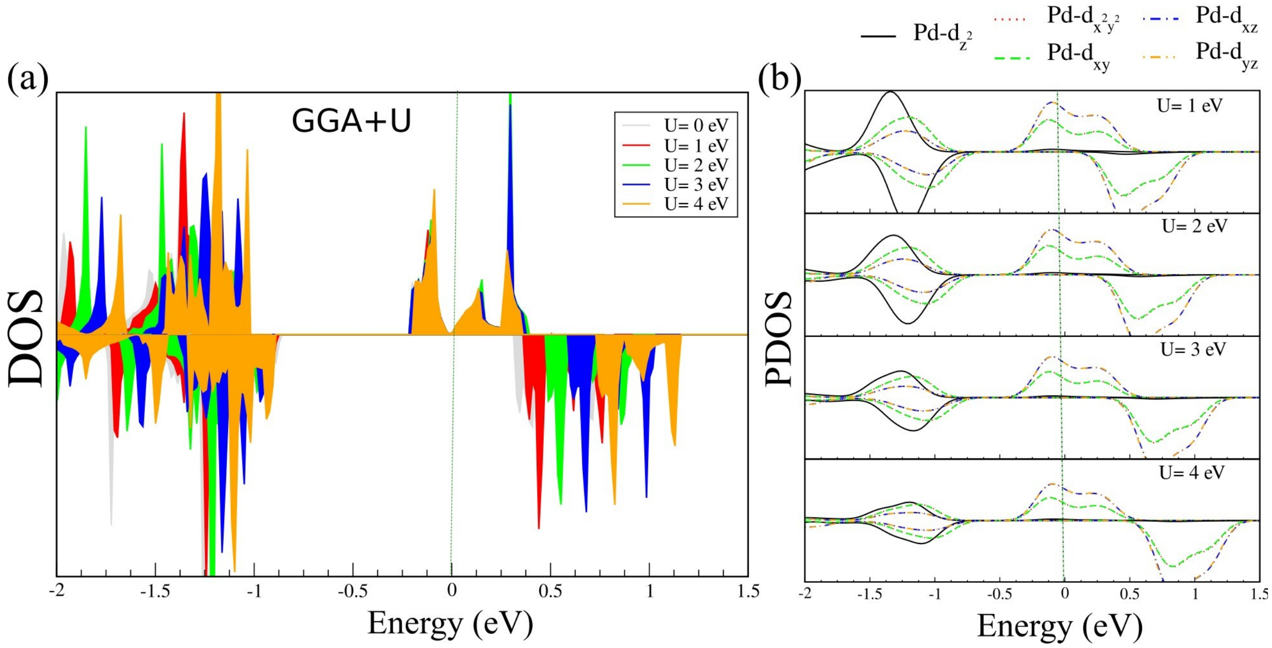

Figure S2: (a) The total DOS of PdCl 3 calculated with the GGA+U for several values of U and (b) the PDOS of PdCl 3 for the Pd d-states with SOC for several values of Hubbard U. The zero of energy is set at E_F .

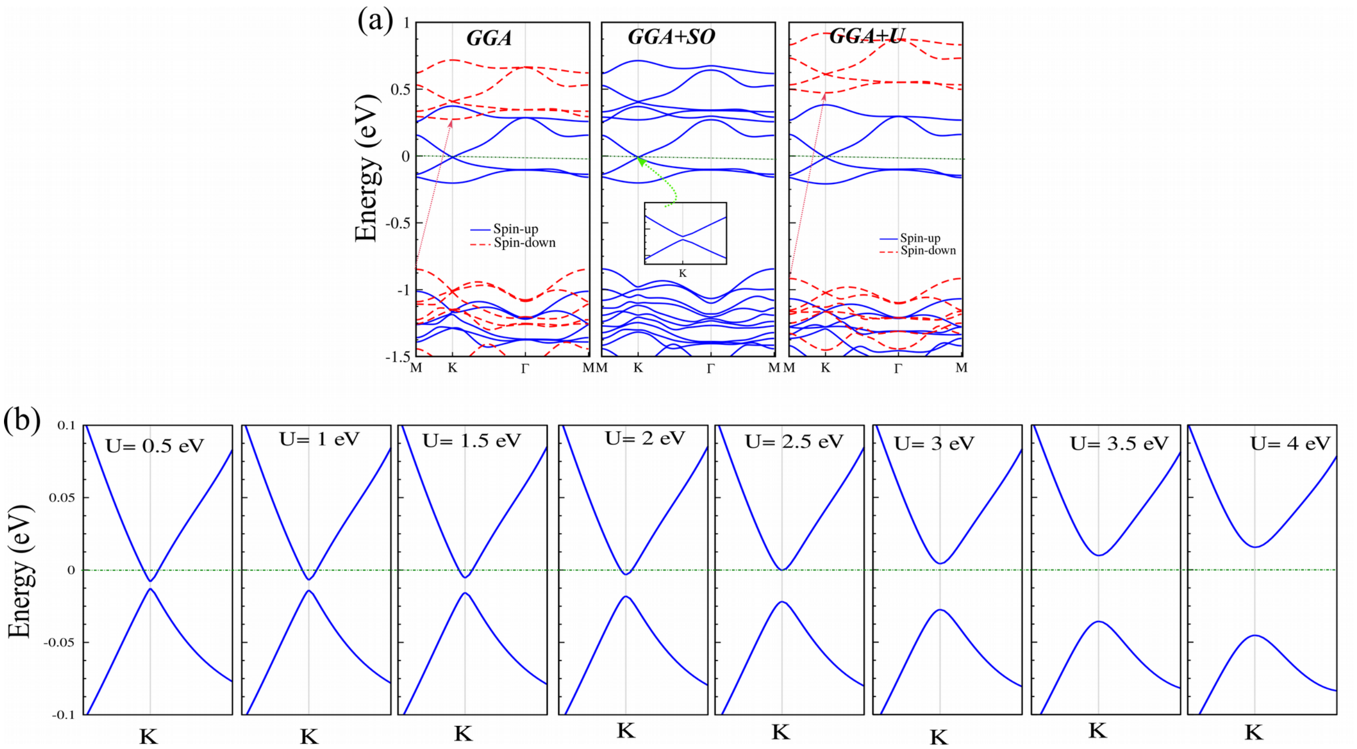

Figure S3: (a) Electronic band structure of PdCl 3 calculated with the GGA, GGA+U, GGA+SO. (b) The band structure in the region of the Fermi level for several different values of Hubbard U.

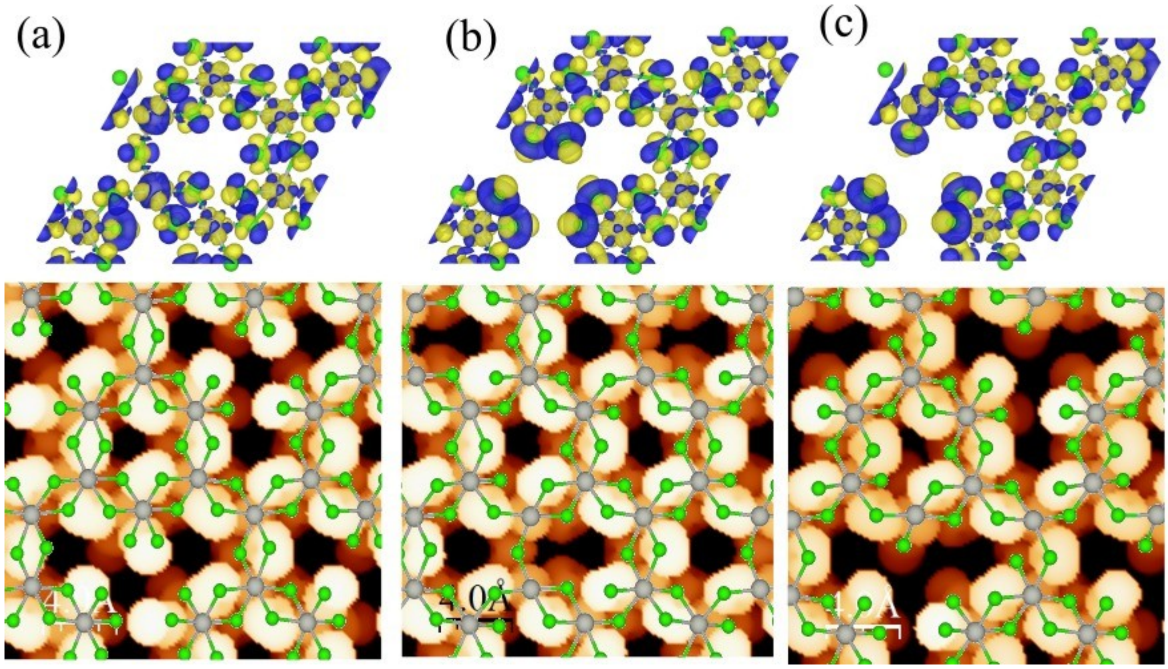

Figure S4: Difference charge densities and simulated STM images for (a) single Pd vacancy, (b) single Cl vacancy and (c) Pd+Cl double vacancy defects. The blue and yellow regions represent the charge accumulation and depletion, respectively. The STM images are overlaid with the $PdCl_3$ atomic structure.

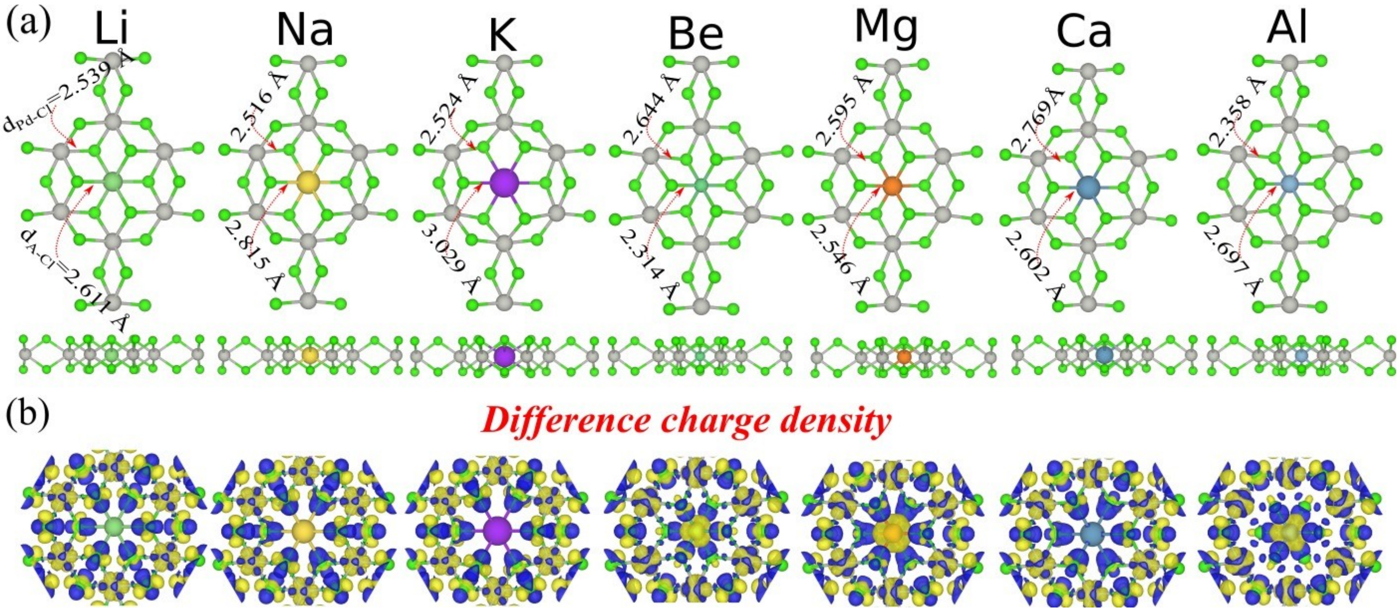

Figure S5: (a) Optimized atomic structures and (b) difference charge densities of adsorbates on $PdCl_3$, namely, Li, Na, K, Be, Mg, Ca and Al adatoms. The blue and yellow regions represent the charge accumulation and depletion, respectively.

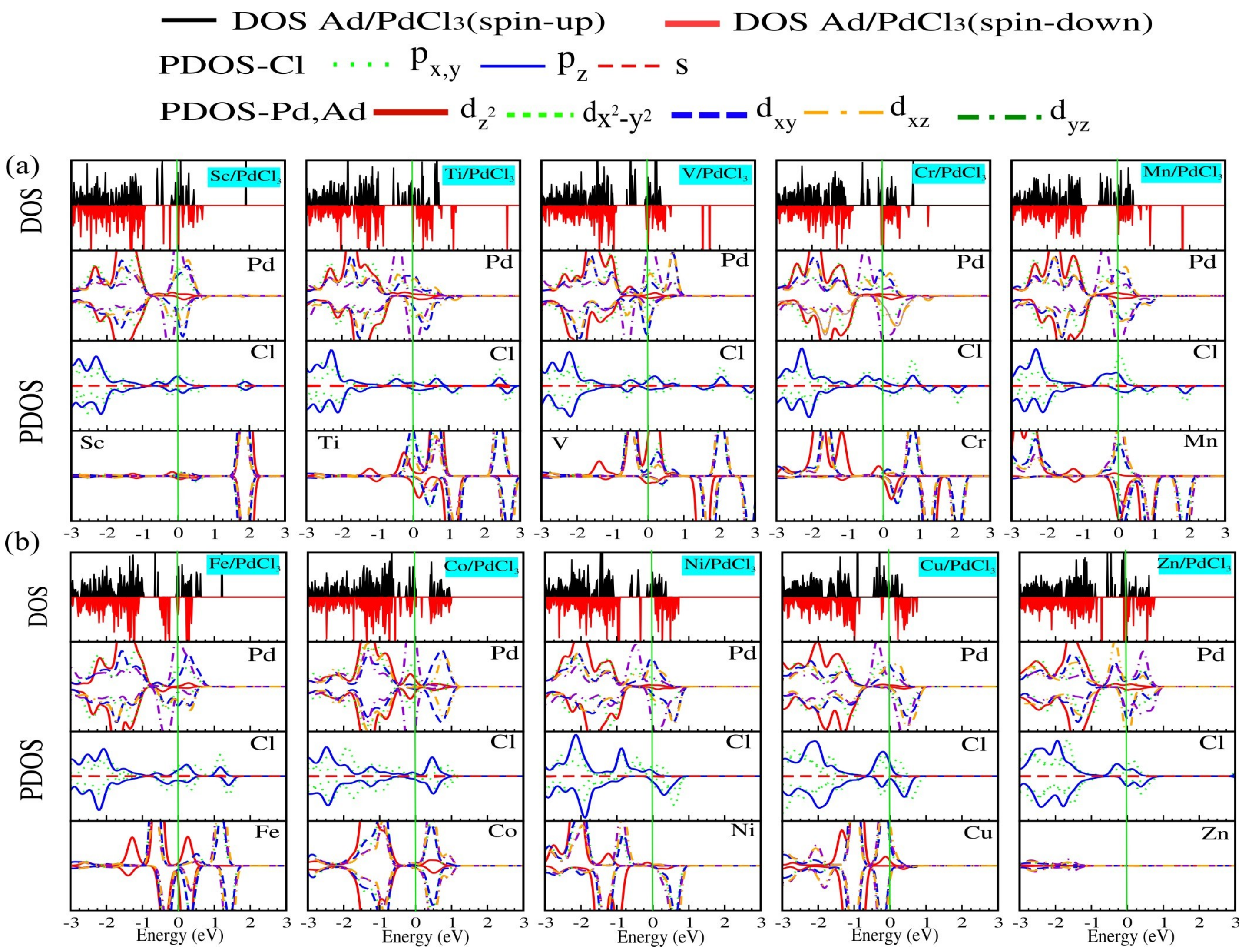

Figure S6: DOS and PDOS of PdCl 3 with adsorbed (a) Sc, Ti, V, Cr, Mn and (b) Fe, Co, Ni and Zn adatoms. The zero of energy is set at E_F .

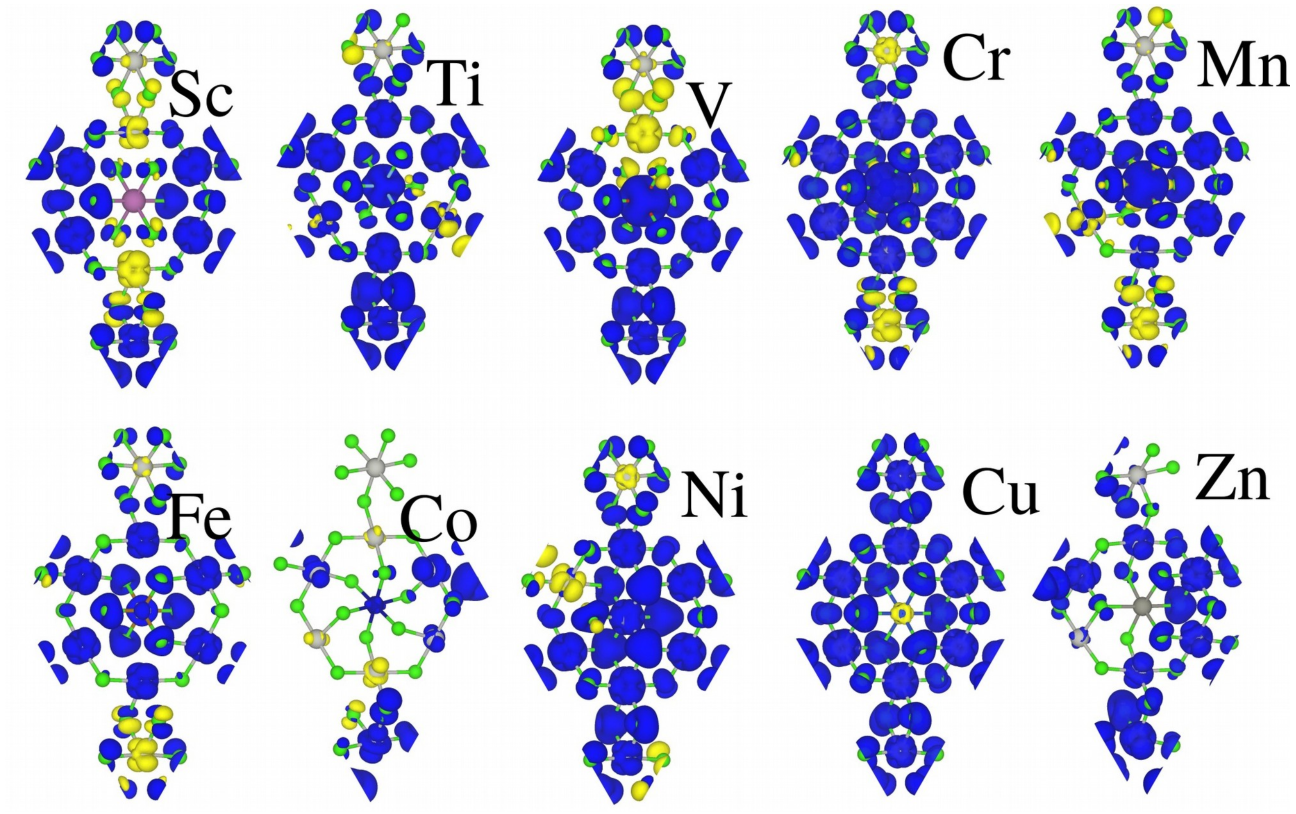

Figure 7: Spin density (difference of the spin-up and spin-down electron density) for the doped $PdCl_3$ systems.

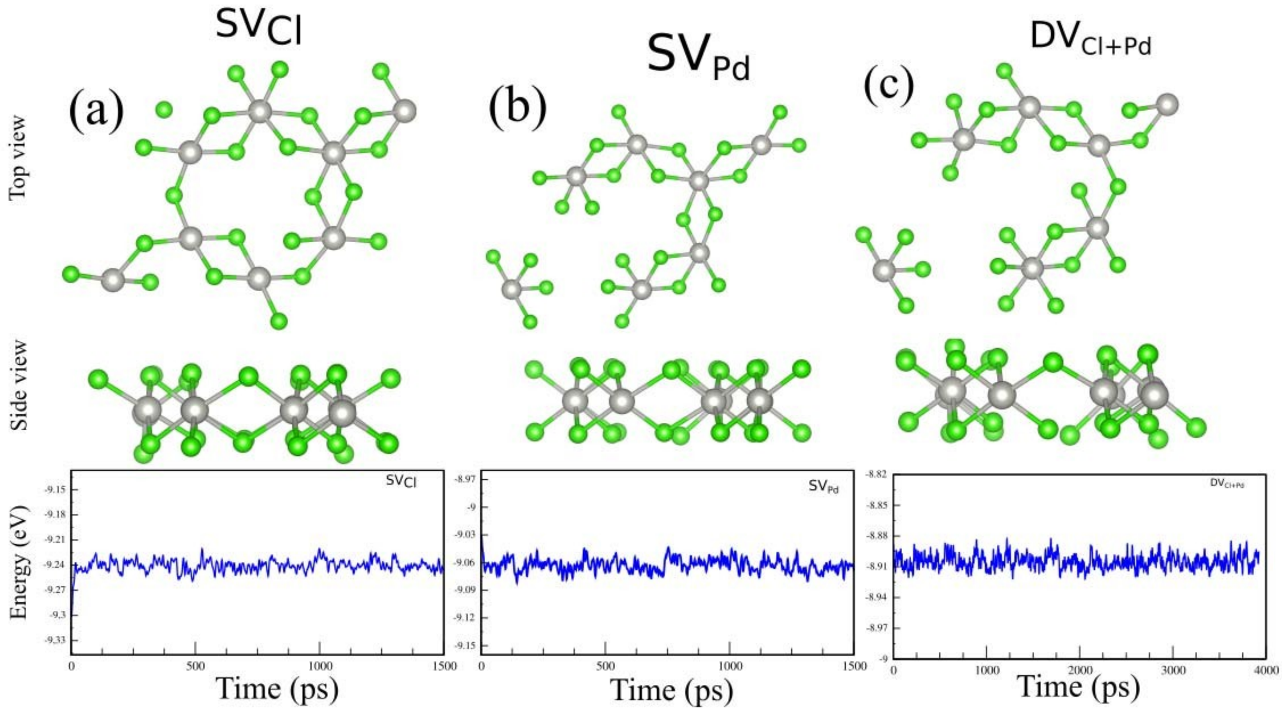

Figure S8: Atomic structures of the single Cl (a) and Pd (b) vacancies, and the double Cl + Pd vacancy (divacancy) (c). The corresponding results for the energy from ab initio molecular dynamics simulations at 300K.

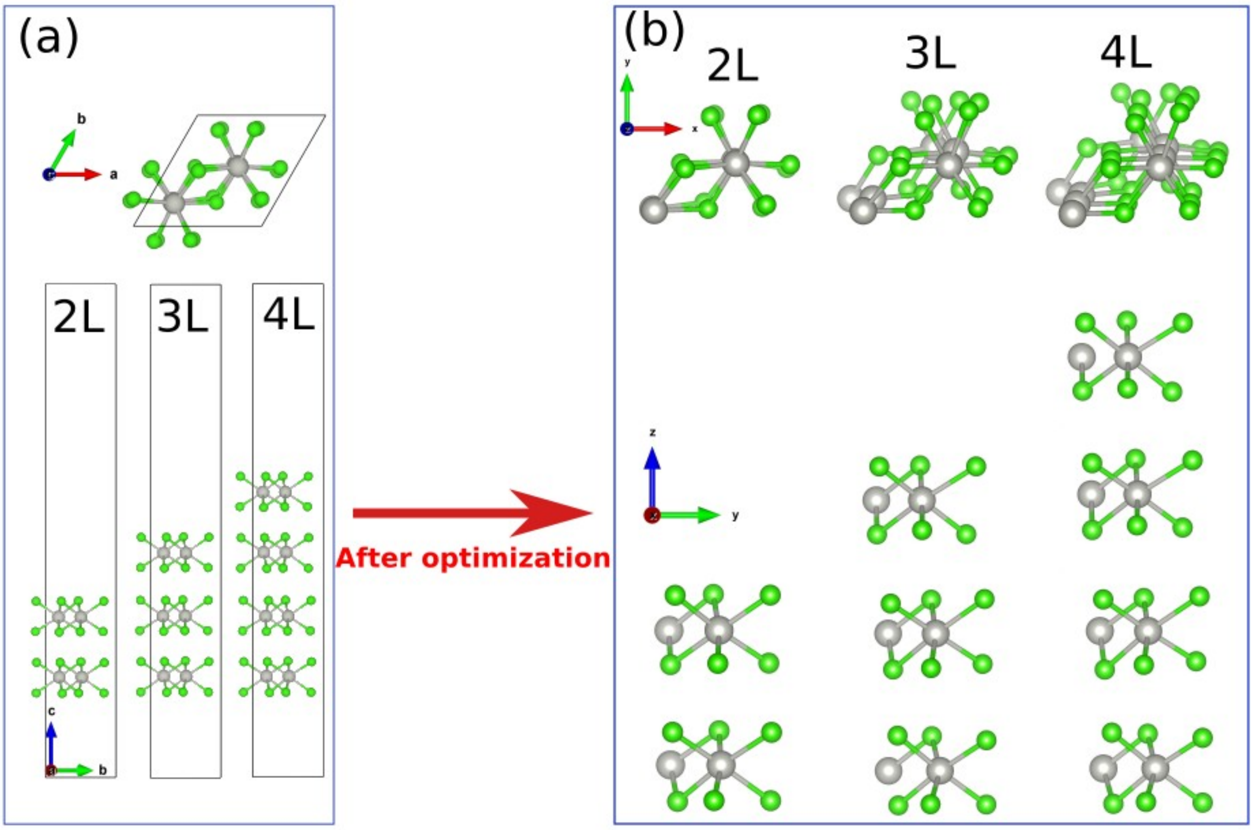

Figure S9: Top and side views of the atomic structurs of few-layer $PdCl_3$: (a) before atomic relaxation and (b) after atomic relaxation.

Table 1: Cohesive energies (eV/atom) from Charles Kittel.Introduction to Solid State Physics,8thedition. Hoboken, NJ: John Wiley and Sons, Inc, 2005; Efthimios Kaxiras.Atomic and Electronic Structure of Solids.Cambridge: Cambridge University Press, 2003. [http://www.knowledgedoor.com/2/elements handbook/cohesive energy.html]

Element	Cohesive energy (eV)
Li	2.03
Na	1.113
K	0.934
Be	3.32
Mg	1.51
Ca	1.84
Al	3.39
Sc	3.90
Ti	4.85
V	5.31
Cr	4.10
Mn	2.92
Fe	4.28
Co	4.39
Ni	4.44
Cu	3.49
Zn	1.35
